# When contexts collide: Spatial context prevails over temporal context in binocular rivalry

**DOI:** 10.1167/jov.25.11.11

**Published:** 2025-09-16

**Authors:** Lisa Beckmann, Thomas Schenk, Karin Ludwig

**Affiliations:** 1 Clinical Neuropsychology, Department of Psychology, Ludwig-Maximilians-Universität München, Munich, Germany

**Keywords:** bistable perception, flash suppression, center–surround interactions, figure–ground segmentation, spatial context cues, temporal context cues

## Abstract

Studying binocular rivalry lets us gain a glimpse into how our visual representation is constructed. It is well-known that what is presented around (spatial) and before (temporal) the rivaling stimuli influences what you perceive. How these spatial and temporal factors interact is, however, unknown. In two experiments, we investigate the influence of the spatial surround and flash suppression (as an example of a temporal effect) on binocular rivalry when they occur concurrently. We used gratings of opposing orientation as rivalry probes and varied the strength of the spatial surround by modulating its contrast and the strength of the flash suppression by modulating either the blank duration (Experiment 1; *n* =17) or the probe duration (Experiment 2; *n* = 17). We measured both the dominant percept and its clarity. For the single context (either spatial or temporal), we could replicate previous findings. Crucially, when spatial and temporal contexts were combined, we could show additive effects when they favored the same grating and opposing effects when they favored different gratings. In these cases, the spatial context exerted a stronger influence than the temporal context. Also, the clarity of reported percepts decreased when influences were conflicting. Exploratory analyses showed surprising differences in perceptual clarity for the different modulations of flash suppression (Experiment 1 vs. Experiment 2), challenging previous assumptions about the interchangeability of blank and probe duration. In general, the results emphasize the importance of looking at different factors shaping bistable perception not just in isolation, but concurrently.

## Introduction

Bistable perception arises when a single, ambiguous stimulus gives rise to two distinct perceptual interpretations that alternate spontaneously over time. This phenomenon offers a rare glimpse into the brain's ability to resolve conflicting information, providing valuable insights into the mechanisms that maintain perceptual stability and facilitate perceptual shifts. By examining bistable stimulus configurations, as well as the conditions under which they are resolved and the factors that bias interpretation in one direction or another, we can deepen our understanding of the processes that bridge visual stimulation and conscious perception. This study focuses on two such factors: spatial and temporal context. To investigate these dynamics, we use binocular rivalry (BR), a well-established method that allows us to explore bistable perception across various stimuli and conditions. In the case of BR, one stimulus (e.g., the image of a face) is presented to one eye, and another stimulus (e.g., the image of a house) is presented to the other at the corresponding retinal location. In BR, subjective perception alternates between the two images for the duration of the stimulus presentation (Brascamp, Klink, & Levelt, 2015; Levelt, 1965). It is well-known that specific features of the stimuli can lead to dominance of one over the other, for example, of the stimulus with higher luminance (Brascamp et al., 2015), higher contrast (Paffen, Tadin, te Pas, Blake, & Verstraten, 2006), or with more contours (Brascamp et al., 2015). When these measures are held constant, the question remains: what else leads to the prioritization of one percept over the other?

### Spatial context gives cues about the most probable perception

Unlike in the laboratory, in the real world, humans never “just see” a house or a face in isolation. These types of stimuli are invariably embedded within a spatial context that provides essential information about the world around us. The house may be surrounded by a garden or stand next to another house.

In an experimental framework, the spatial context is often provided in the form of a surrounding annulus around a central (ambiguous) test stimulus, which results in a center-surround interaction of the two (Carter, Campbell, Liu, & Wallis, 2004; Ichihara & Goryo, 1978; Mapperson & Lovegrove, 1991; Paffen, te Pas, Kanai, van der Smagt, & Verstraten, 2004). Previously, we confirmed earlier results (Carter et al., 2004; Fukuda & Blake, 1992; Ichihara & Goryo, 1978; Mapperson & Lovegrove, 1991; Ooi & He, 2006; Paffen et al., 2006; Paffen, Alais, & Verstraten, 2005; Paffen, van der Smagt, Pas, & Verstraten, 2005; Sereno & Sereno, 1999; Wook & Shevell, 2008), namely, that the surrounding spatial context is used to disambiguate the rivaling stimulus (Beckmann, Schenk, & Ludwig, 2025). In cases of ambiguous grating stimuli, of which one matches the surrounding grating (in orientation) and one has a different orientation, participants tend to see the non-matching grating, for example, the clockwise rotated grating, when surrounded by a counterclockwise rotated grating. We further found that this effect was irrespective of the ambiguity of the surround and was reduced in strength when the surround was at a low contrast or reduced in size (Beckmann et al., 2025). This effect is assumed to rely both on the suppression of the central grating that matches the surround via surround inhibition and the facilitation of the grating that differs from the surround via both lateral inhibitory and facilitative connections (Blakemore & Tobin, 1972; Jones, Grieve, Wang, & Sillito, 2001; Knierim & Van Essen, 1992; Sillito, Grieve, Jones, Cudeiro, & Davls, 1995). In the real world, however, context is not just a static two-dimensional image. The visual environment changes across time and visual perception is determined both by spatial and temporal changes.

### Temporal context influences perception

Information from cues presented before a test stimulus can also be used to clarify perception. For instance, the dominant percept during a BR display influences the perception of a following BR display. The duration between the offset of the first BR display and the onset of the second BR display modulates the direction of the influence. Shorter gaps (<500 ms) tend to cause a transition from one percept to another, whereas longer gaps (>500 ms) cause the impression of the same perception as before the gap (Brascamp, Pearson, Blake, & van den Berg, 2009; Klink et al., 2008; Pearson & Brascamp, 2008; Sterzer & Rees, 2008). The influence is believed to be based on a type of perceptual memory (Pearson & Brascamp, 2008).

Another well-known paradigm using temporally presented cues is flash suppression (FS), which can be considered a special case of BR. Here, a stimulus (e.g., a horizontal grating), is presented to one eye for a short amount of time (prime duration). Then, the stimulus vanishes very briefly (blank duration). Finally, two competing images are shown to the eyes for a certain time interval (probe duration), in our example, the horizontal grating to the same eye as the prime and a vertical grating to the other. In FS, the new stimulus (the flash) typically dominates perception, which is, in this case, the vertical grating. The probe stimulus (the horizontal grating) already shown during the prime is suppressed. However, the observed perceptual effects depend on different temporal variables, such as the prime, blank, and probe durations (see Supplementary Table S1 for an extensive overview of the FS literature). For instance, prolonging the blank duration, that is, the time no stimulus is shown in between, reduces the suppression effect (e.g., Experiment 1 in Wolfe, 1984). The duration of the presentation of the prime stimulus also affects what is perceived in the later binocular configuration—short prime durations favor the perception of the pattern presented in the prime stimulus and long prime durations favor the perception of the novel pattern during the probe (Brascamp, Knapen, Kanai, van Ee, & van den Berg, 2007). Sometimes, probe and blank durations are manipulated together, which Ikeda et al. referred to as stimulus offset asynchrony (SOFFA) (Ikeda, Ogata, & Morotomi, 2009; Ikeda & Morotomi, 2002, 2007). Similar to manipulating the blank duration, an increase in the length of the SOFFA leads to a decrease in the suppression effect (Ikeda et al., 2009; Wolfe, 1984).

Different ideas of the mechanism behind FS have been suggested. Wolfe (1984) rejected the notions of forward masking, monocular fatigue, and neuronal adaptation. Instead, he suggested that FS raises the threshold for stimulation of the primed eye (Wolfe, 1984). More recent studies revisit neuronal adaptation (Brascamp et al., 2007; Holmes, Hancock, & Andrews, 2006; Ikeda & Morotomi, 2002) as an explanation and criticize that Wolfe (1984) only used an intraocular prime, did not investigate interocular transfer or feature selectivity, and therefore could not exclude neuronal adaptation (Ikeda & Morotomi, 2002). In a second study, Ikeda and Morotomi (2007) presented a homogenous gray field as a prime stimulus and blue and red rivaling stimuli as the probe. They found that the eye that was not previously stimulated dominated perception. Thus, the authors suggested that FS does not need specific features to occur and can be purely eye-of-origin based. However, it is not independent of features when they are present (Ikeda & Morotomi, 2007). Brascamp et al. (2007) described that both flash suppression and facilitation exist and result from the same underlying neuronal process but occur in different situations, that is, FS for long high-contrast prime durations and flash facilitation for short prime durations and low-contrast prime stimuli.

## Experiment 1

Both spatial and temporal context cues have been shown to influence bistable perception. However, we must consider that, in everyday vision, spatial and temporal contexts occur together and, on some occasions, might yield conflicting influences. Therefore, we conducted an experiment testing the effects of both temporal context (in the form of FS) and spatial context (in the form of surround influences [SI]) to see how they would interact and to determine which context would exhibit the stronger influence. As detailed in the previous section, in most cases of FS, the temporal context pushes the stimulus non-matching to the prime stimulus (and/or eye) to dominate perception. In SI (spatial), the non-matching grating is also expected to dominate perception.

To ensure that our experimental settings did not inherently favor one type of context in general, we used conditions that varied the strength of each type of context: SI: strong: high contrast, weak: low contrast; FS: strong: short blank duration, weak: long blank duration, that is, decreasing strength with increasing blank duration: 0, 120, 250, and 500 ms. In the FS literature, there are two ways in which responses are retrieved: either as a forced choice query or a rating describing the participant's percepts. We decided to use the more common and straightforward way—a two-alternative forced choice (2AFC) query (see Supplementary Table S1). Because participants are obliged to choose a percept in a 2AFC paradigm, the information about how easy the choice was is lost, and thereby information about the phenomenology of their percept. Hence, we added a perceptual clarity rating after the 2AFC choice to be able to reconstruct this phenomenological aspect further. Our rating scale was adapted from the perceptual awareness scale (Ramsøy & Overgaard, 2004) for our purposes.

We first sought to replicate previous findings: FS predicts that the new stimulus will gain dominance. As the blank duration increases, the strength of this effect typically decreases. Similarly, we wanted to replicate the inhibitory effect of a surrounding annulus. We expect this effect (dominance of the non-matching grating) to be stronger when the annulus has a high contrast (90%) compared with a low contrast (10%).

When combined into a single paradigm, the spatial surround will be presented around the probe (of the FS part of the experiment). This probe will thus be influenced by the preceding stimulus (the FS prime: temporal) and the concurrent stimulus (the SI surround: spatial).

We expect the effects to work together in an additive manner when the influences on the probe percept are the same. More important, when the influences go in different directions, we want to see whether a spatial or a temporal context takes priority in influencing perception. Because we are the first research group to combine spatial and temporal contexts within the same bistable paradigm, it is difficult to predict a priori which context would provide the stronger cues. Thus, this hypothesis remains without direction. Regarding our clarity rating, we expect it to be higher in additive than in competitive trials.

### Methods

#### Participants

A total of 17 participants (8 females and 9 males) took part in this experiment. They were recruited from the Ludwig-Maximilians-Universität München and the Technische Universität München, as well as from outside the universities. Compensation for their time was provided as 10€ per hour or course credit. Ages ranged from 19 to 40 years, with an average age of 20.12 ± 4.77 years.

An a priori power analysis was conducted to determine the required sample size. Two pilot studies (*N*_Pilot1_ = 6 and *N*_Pilot2_ = 7) were conducted to estimate effect sizes for both spatial and temporal effects. The weakest observed effect, temporal effect in pilot 2: t(6) = 2.31, *p* = 0.037, mean difference = 0.18 ± 0.20, was used to calculate the required sample size. Using a paired-sample *t*-test (one-sided) and setting alpha to 0.05 and power to 0.90, the minimum required sample size was determined to be at least 13 participants. We decided to recruit 17 participants in case some participants needed to be excluded.

We only included right-handed participants without a stated psychological or neurological disorder. All participants had normal or corrected-to-normal visual acuity. They used their right hand to report their perceptual judgment and their left hand to indicate the clarity rating. All participants gave informed written consent before participating. The local ethics board of the Department of Psychology of the Ludwig-Maximilians-Universität München approved the study.

#### Apparatus

The stimuli for this experiment were generated with Matlab R2018a using the Psychtoolbox version 3.0.16 (The MathWorks Inc., 2018) on an HP EliteDesk 800 G3 TWR computer. An AOC LCD monitor (27 inches, 100-Hz refresh rate) was used to present the stimuli. Through a double mirror stereoscope, participants saw part of the right half of the screen with the right eye and part of the left half of the screen with the left eye. The distance between the monitor and the participant's eyes (including distances within the mirror stereoscope system) was 56 cm.

#### Stimuli

**Figure 1.**
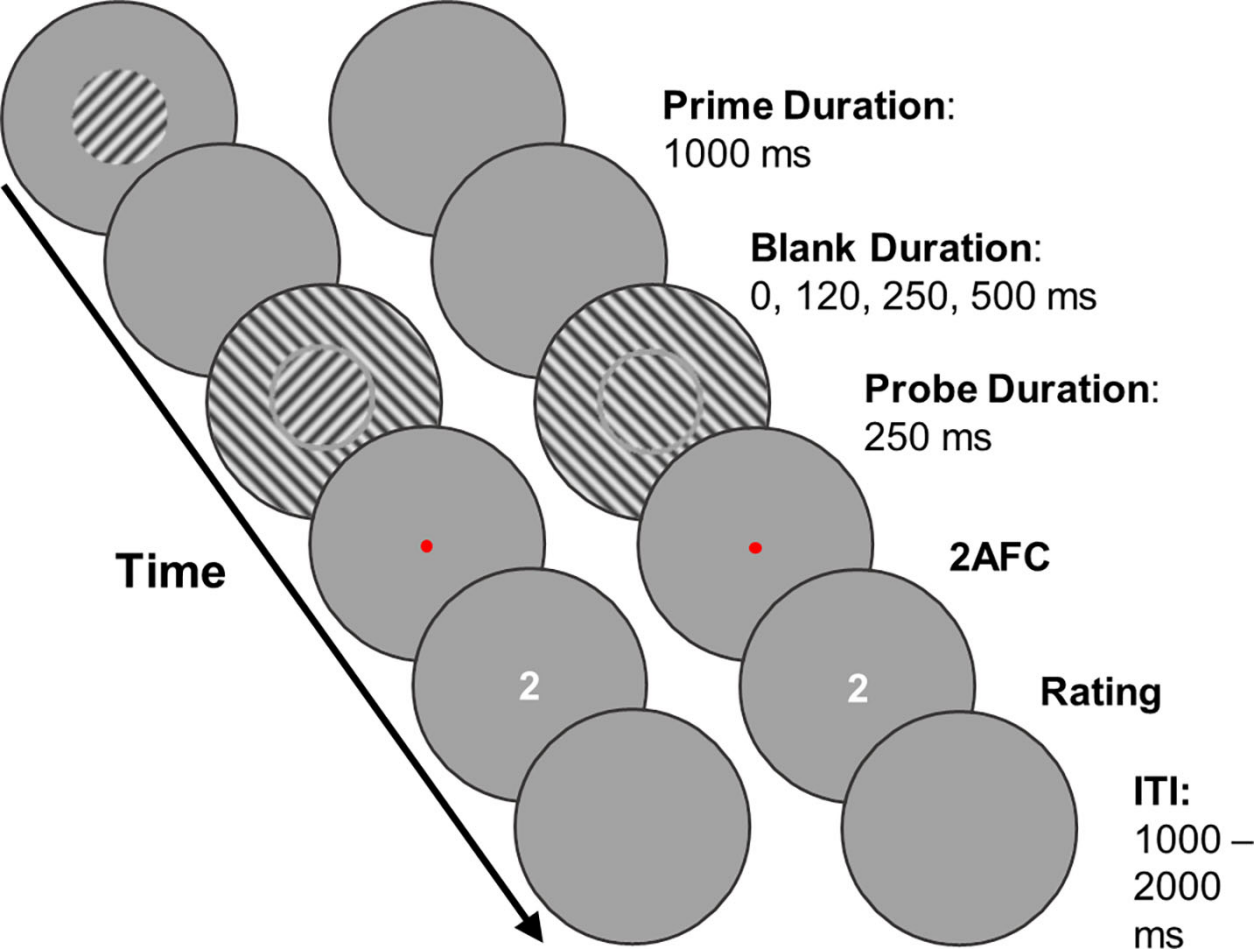
This figure shows an example trial of Experiment 1 with both temporal and spatial context; the left circles represent what was presented within a circular fusion frame to the left eye, and the right represents the presentation to the right eye. Participants first saw a prime stimulus for 1,000 ms, followed by a blank duration varying between 0, 120, 250, and 500 ms, then a probe for 250 ms. In the experimental condition, during the probe presentation, both eyes saw a rivalry target (CW in one eye, CCW in the other eye) that was surrounded by a grating, completing only one of the patterns of the rivalry targets. The probe was followed by a 2AFC prompt: a red fixation dot that was present until the participant pressed one of the response keys. Afterward, participants provided their rating of perceptual clarity. A rating between 0 and 3 could be selected (0 [unclear] to 3 [maximal clarity]) using the up- and down-keys and confirmed with the space bar. This action marked the end of the trial. The interval between trials (ITI) lasted between 1,000 and 2,000 ms.

The stimuli consisted of a central sinusoidal grating and, in some conditions, a surrounding grating. The central grating was ambiguous (i.e., rivaling grating presented to the two eyes) when the stimulus was presented as a probe. The gratings were rotated 45 degrees clockwise (CW) or counterclockwise (CCW) from the vertical. The complete stimuli, central grating, and surround extended 1.5 degrees of visual angle (dva), with the central grating per se extending to 0.83 dva, which is small enough to reduce patchwork perception to a minimum at our spatial frequency (O'Shea, Sims, & Govan, 1997). The gratings were at either 90% (high-contrast condition) or 10% Michaelson contrast (low-contrast condition). The rivalry target and the spatial surround were separated by a very small gray gap to indicate to the participant for what part of the stimulus they were supposed to report their perception (Figure 1). The stimuli were surrounded by a circular dark gray frame with black circles included to reinforce binocular fusion (the stimulus size with fusion frame was 3.7 dva). Stimuli presented on the left side of the screen were visible to the left eye only (through the left port of the double mirror stereoscope), and stimuli presented on the right side to the right side only. The grating had a spatial frequency of 8.43 cycles/dva. The mean luminance of all gratings at the position on the screen was 100 cd/m^2^ at high contrast and 101 cd/m^2^ at low contrast. When there was no surround, the rivalry target was surrounded by a gray ring with the same luminance as the central target [RGB left: 159, RGB right: 163]. Stimuli were presented on a gray background. The luminance of the gray background corresponded with the average grating luminance.

#### Procedure

Participants were first asked to adjust the stimulus positions onscreen until they achieved fusion of the images presented to the two eyes. They were then asked to complete 12 practice trials. These trials included examples of all relevant combinations (high- and low-contrast surround, all blank durations, and all baseline conditions). If participants struggled to report a clear percept, they were asked to repeat the practice. Each trial (Figure 1) started with a prime stimulus presented monocularly for 1000 ms, as commonly used (Supplementary Table S1).

After the prime stimulus presentation, a blank frame followed, with durations varying between 0, 120, 250, and 500 ms. Subsequently, participants viewed probe stimuli for 250 ms, consisting of a central grating with or without a surrounding annulus. They then reported their percept using the right hand (left arrow key = CCW, right arrow key = CW) and provided a perceptual clarity rating using the up/down arrow keys (default = 2). Responses were logged with a space bar press. A schematic of the trial sequence is shown in Figure 1.

The clarity rating had been explained to them in the instructions. Resembling the perceptual awareness scale (Ramsøy & Overgaard, 2004), they were told to rate according to the following rules: 0 = “The percept was 50/50 and I might as well have reported the other,” 1 = “There was a small tendency for the reported direction, but the other was also relatively strong,” 2 = “It was relatively clearly this percept but I also saw some of the other,” and 3 = “I only saw this grating.” The subsequent trial started after an intertrial interval of 1,000 to 2,000 ms.

**Figure 2.**
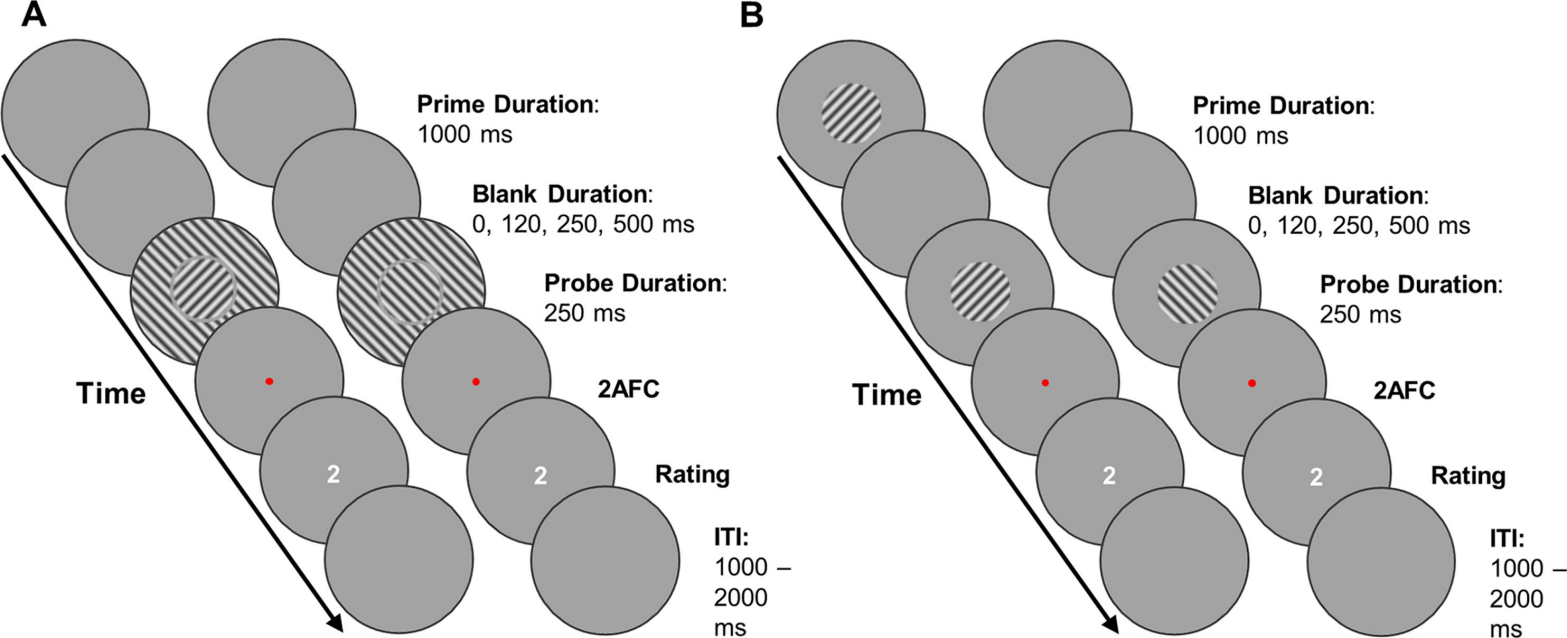
Example baseline trials of Experiment 1. Each circle represents what was shown to each eye within a fusion frame. (**A**) SI baseline condition. No stimulus was shown during the prime interval in the SI baseline condition. The durations for all intervals were identical for the SI baseline and the experimental conditions. During the probe interval, participants saw two central gratings (rivalry target) that were rotated CW or CCW. These gratings were surrounded by an annulus that completed one of the gratings. The probe was followed up with a 2AFC task to determine which grating was dominant, indicated by a red fixation dot, and presented until a keypress. Afterward, participants logged a perceptual clarity rating between 0 and 3. Each trial ended in an ITI randomly ranging between 1,000 and 2,000 ms. (**B**) FS baseline condition. In this condition, participants did see a prime. Apart from the absence of a surrounding annulus during the probe interval, everything was the same as in the experimental conditions.

An SI baseline condition allowed us to compare the experimental conditions against an SI effect free of temporal context (Figure 2A). Similar to the experimental condition, we had prime and blank durations. However, nothing was presented at the prime duration (Figure 2A). This approach ensured that all trials, including the SI baseline, were of comparable lengths, maintaining a consistent flow for the participants without disrupting their responses. Similarly, we created an FS baseline without any pattern surround, i.e., the procedure was the same as in the experimental condition; however, no surround was present during the probe duration (Figure 2B).

Our main independent variables are the following: 1) FS: blank duration: the duration between prime and probe (0 ms, 120 ms, 250 ms, and 500 ms, times that vary the strength of FS according to Supplementary Table S1), and (2) SI: surround contrast: contrast of the annulus (10% and 90%). Additionally, we have three further variables that need to be controlled for 3) tilt of the prime stimulus grating (CW and CCW), 4) eye viewing the prime stimulus (left and right), and 5) tilt of the surrounding annulus grating (CW and CCW). All variables were randomized. Participants completed 8 blocks of 56 trials each, yielding a total of 448 experimental trials; this took participants between 60 and 90 minutes to complete.

#### Ensuring luminance uniformity

Differences in luminance were accounted for by creating two lookup tables with gamma-corrected information to ensure equal luminance for each side of the monitor. This correction ensured that all stimuli were equally bright regardless of their position on the monitor; thus, stimulus position differences were not confounded by luminance differences.

#### Analyses and data preprocessing

Given the binary nature of the main outcome variables, that is, whether the predicted (1) or the unpredicted pattern (0) was reported in each trial, we used generalized linear mixed models (GLMMs) with a binomial error distribution and logit link function to analyze the occurrence of percepts. For the analysis of clarity ratings, which were collected on an ordinal scale, we used cumulative link mixed models (i.e., ordinal logistic regressions) to account for the ordered categorical nature of the responses. All models included participant as a random intercept to account for repeated measures across trials, and fixed effects corresponding with the experimental manipulations relevant to each hypothesis (e.g., blank duration, surround contrast, and trial type).

Model comparisons were conducted using likelihood ratio tests (LRTs) and by examining changes in the Akaike information criterion (AIC) to assess the contribution of each fixed effect. McFadden's pseudo R² was reported as an effect size for LRTs comparing nested models. Importantly, R²_McFadden_ is a pseudo R², not the standard R². Because R²_McFadden_ measures improvement in log-likelihood relative to a baseline model (here, the intercept-only models) rather than explained variance, values tend to be considerably lower. McFadden himself describes values between 0.2 and 0.4 as representing an excellent fit (McFadden, 1973). When comparing model fits, we often reported the ΔR²_McFadden_, which is the difference between R²_McFadden_ for the two models tested and thus represents the improvement in fit, not the fit per se. Where appropriate, planned contrasts were tested using estimated marginal means, adjusted for multiple comparisons using Bonferroni correction. In some cases, Wald tests were used to assess the significance of model intercepts, particularly to determine whether the predicted probability of a percept deviated from chance level (i.e., 50% in 2AFC tasks).

For competitive trials, where spatial and temporal context cues predicted opposing percepts, responses were coded in line with SI predictions, and statistical models tested the influence of both factors and their interactions. To assess the relative strength of FS and SI under matched conditions, we additionally conducted paired Bayesian *t*-tests on baseline conditions to identify perceptually equivalent combinations. Then we compared the identified comparisons under competitive conditions via further Bayesian *t*-tests. Raw data, as well as all analyses, can be found online (https://osf.io/q4zfn/).

### Results

#### Baseline validation

The findings from the baseline conditions served two goals. First, we needed the data from the baseline conditions as reference values for the experimental conditions. Second, we wanted to verify whether our implementation of the respective paradigms reproduced the findings in the relevant literature (Beckmann et al., 2025; Wolfe, 1984). In the case of the FS paradigm, participants saw a central grating during the prime, which vanished for varying amounts of time (blank duration). At the probe presentation, two rivaling gratings were presented without any surround. In line with Wolfe's (1984) results, we expected that the dominant percept during the probe was, in most cases, opposite to the pattern presented during the prime interval. To statistically evaluate this tendency and the potential influence of blank duration on the perception outcome, we fitted GLMMs with a binomial response (percept in line with FS vs. not in line with FS) and participant as a random intercept.

First, we tested a model with only a random intercept for participant. A Wald test on the resulting intercept indicated that, across blank durations, participants were significantly more likely to perceive the central grating that was different from the prime grating than the one which was identical to the prime grating (β = 1.06, standard error [SE] = 0.21, Z = 4.99, *p* < 0.001, 95% confidence interval [CI] 0.64–1.47), thus demonstrating the basic FS effect in our data.

**Figure 3.**
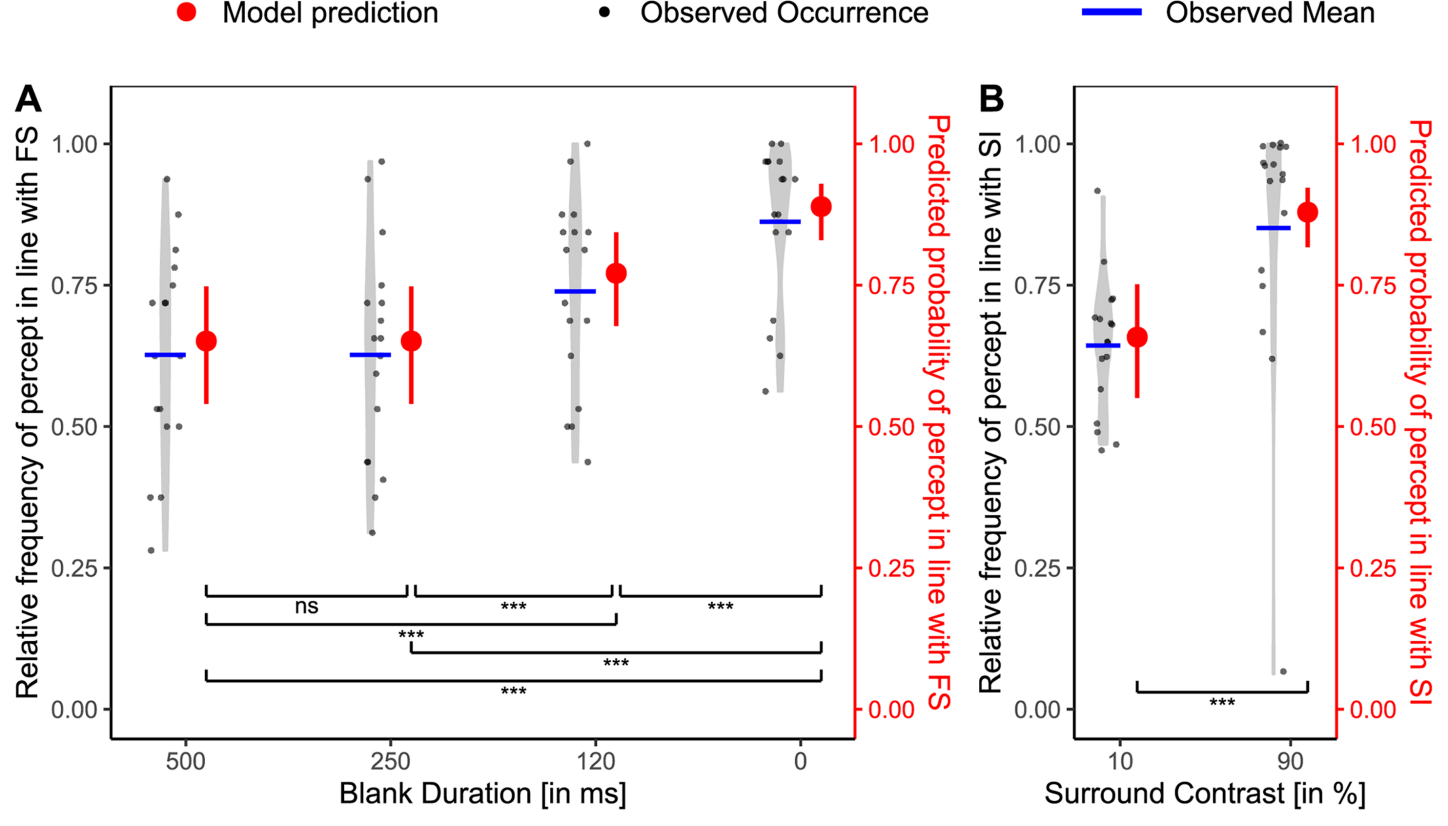
Baseline condition results for FS (**A**) and SI (**B**) in Experiment 1. (**A**) Relative frequency of the percept reported in line with FS paradigm (black, left *y* axis) per participant and corresponding model predictions (red, right *y* axis) across varying blank durations for the FS baseline condition. Blank durations are ordered on the *x* axis from weakest hypothesized FS effect (500 ms) to strongest (0 ms). Light gray violin plots represent the distribution of individual data, with jittered black dots indicating the observed values, and blue horizontal lines showing the observed condition means. Red points with error bars show model-predicted means and their 95% CIs. Statistical significance is based on Bonferroni-corrected planned comparisons of model predictions. ns, not significant. *p_adj_ < 0.05; **p_adj_ < 0.01; ***p_adj_ < 0.001. (**B**) Same as (**A**), but for the SI paradigm. Surround contrasts are ordered from weak (10%) to strong (90%) in terms of hypothesized SI strength. All visual conventions from (**A**) apply.

Second, we sought to replicate results from Wolfe (1984), who showed an effect of blank duration on the strength of FS. More specifically, as the blank duration decreased, the FS effect increased. For our data, we could confirm this pattern. To assess the effect of blank duration, we compared the random-intercept model with an extended model including blank duration as a fixed effect. As a reference value, we used the hypothesized weakest condition, which was the 500-ms blank duration. The model including blank duration provided a significantly better fit than the random-intercept model. This was suggested both by the LRT, χ^2^(3) = 119.27, *p* < 0.001, R²_McFadden_ = 0.049, and by the lower information criterion for the model with blank duration (AIC = 2314.51) as compared with the random intercept model (AIC = 2427.78). Pairwise comparisons were conducted using estimated marginal means, adjusted for multiple comparisons using the Bonferroni method. Except for the difference between 500 and 250 ms, all pairwise differences were significant, indicating, together with Figure 3A, that shorter blank durations result in a stronger effect of FS. These results are in line with findings by Wolfe (1984).

Similarly, we also implemented a baseline for the SI effect. Here, participants saw nothing until the probe stimulus (two rivaling gratings) appeared. During the probe interval, the central gratings were surrounded by a non-ambiguous surrounding grating which could have a high or a low contrast. Here, we investigated whether the stimulus different from the surround grating would dominate perception (Beckmann et al., 2025) and whether this effect was modulated by the surround contrast (Beckmann et al., 2025; Paffen et al., 2006). We first fitted a random intercept model, that is, one that included only the random factor of participant, to the SI baseline results. A Wald test on the resulting intercept indicated that, across the two contrast conditions, participants generally perceived the central grating whose orientation differed from the surround, β = 1.21, SE = 0.21, Z = 5.84, *p* < 0.001, 95% CI 0.80–1.62.

To assess whether surround contrast modulated this effect, we fitted a second model that included the contrast as a fixed effect, with the weaker condition (10% contrast) as the reference level. This model significantly improved the fit compared with the random-intercept model, as indicated by a significant LRT, χ^2^(1) = 72.63, *p* < 0.001, R²_McFadden_ = 0.063, and a lower AIC for the model including contrast (AIC = 1077.25), compared with the random intercept model (AIC = 1147.88). The fixed effect for high surround contrast was significant and positive (β = 1.33, SE = 0.16, z = 8.10, *p* < 0.001). Because the model is on the logit scale and the intercept reflects the 10% contrast condition (β = 0.66, SE = 0.23, *p* = 0.004), this result indicates that participants were significantly more likely to perceive the central grating that differed from the surround when the surround contrast was high (Figure 3B).

#### Spatial and temporal contexts are additive

There were conditions where SI (spatial context) and FS (temporal context) made identical predictions about the participants' perception. An example of this condition is when the surround annulus grating was rotated CCW and the prime stimulus was a CCW grating. Both FS and SI would predict a CW percept during the presentation of the probe. Accordingly, we expected that perceptual biases seen in the FS baseline conditions would be amplified by adding a surround, more moderately with a low-contrast and more strongly with a high-contrast surround. Similarly, this should be the case when adding an FS prime before our basic center-surround stimulus: The shorter the FS blank duration between prime and center-surround stimulus, the stronger the additive effect (compared with the SI baseline) was expected to be.

To investigate how much adding a surround at different surrounds would boost predictions, in the first analysis, we compared trials in which FS and SI were hypothesized to exert effects in the same directions with the FS baseline trials. First, we fitted a pure random intercept model (random factor participant) as a basis for model comparison. Then we fitted a model with an added additivity factor with three levels: FS baseline, FS baseline + 10% surround contrast, and FS baseline + 90% surround contrast. This improved the fit significantly, as demonstrated by the LRT, χ^2^(2) = 73.22, *p* < 0.001, R²_McFadden_ *=* 0.017, and a decrease in the AIC (ΔAIC = 69.22).

**Figure 4.**
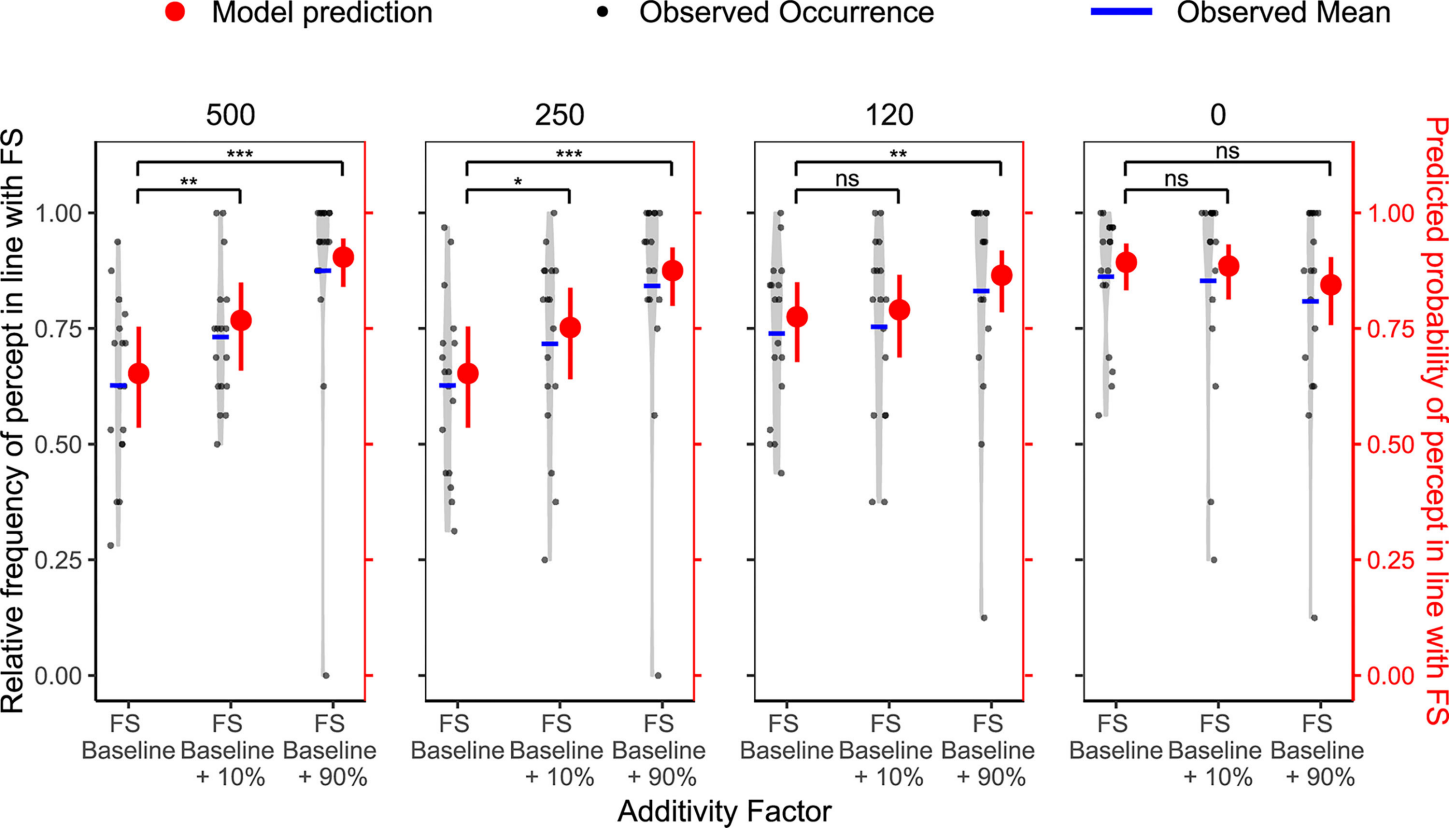
Additivity of surround contrast and temporal context in Experiment 1. Relative frequency of the percept reported in line with the FS paradigm (black, left *y* axis) per participant and corresponding model predictions (red, right *y* axis) across different levels of the additivity factor. The *x* axis is ordered from weakest to strongest, starting with the pure FS baseline to FS baseline with the weaker SI condition (10%) and then with the strongest SI condition (90%). Light gray violin plots represent the distribution of individual data, with jittered black dots indicating the observed values, and blue horizontal lines showing the observed condition means. Red points with error bars show model-predicted means and their 95% CIs. Statistical significance is based on Bonferroni-corrected planned comparisons of model predictions. Ns, not significant. *p_adj_ < 0.05; **p_adj_ < 0.01; ***p_adj_ < 0.001.

Next, we modeled the effect of blank duration alone and found a significantly better fit than the random intercept model, χ^2^(3) = 88.81, *p* < 0.001, R²_McFadden_ *=* 0.020, ΔAIC = 82.81. Combining both predictors (additivity factor and blank duration) in a single model further improved the fit relative to the simple additivity factor model, χ^2^(3) = 90.54, *p* < 0.001, ΔR²_McFadden_ *=* 0.021, ΔAIC = 84.54, and relative to the simple blank duration model, χ^2^(2) = 74.95, *p* < 0.001, ΔR²_McFadden_ *=* 0.017, ΔAIC = 70.95. Crucially, we also found a significant interaction between the additivity factor and blank duration, χ^2^(6) = 57.39, *p* < 0.001, ΔR²_McFadden_ *=* 0.013, ΔAIC = 45.39. The interaction revealed that the surround had an additive effect on top of the FS effect when the FS effect was weak (strong additivity for 500 and 250 ms blanks; weak additivity for 120 ms blanks), but not when the FS effect was strong (in the 0 ms blank condition) (Figure 4).

The mirror analysis (Supplementary Material S2), investigating the effect of adding FS to SI, yielded similar results: in the weak SI condition (10% contrast), there was additivity (Supplementary Figure S1, left). In the strong SI condition (90% contrast), the SI effect already had such a strong effect on perception that adding FS—even strong FS conditions—did not further tip perception toward the predicted grating (Supplementary Figure S1, right).

#### Spatial context overrules temporal context

Next, we tested how participants resolve perceptual ambiguity when spatial and temporal contexts provide opposing predictions. For example, when the surround annulus grating is rotated CCW (CW percept predicted by SI), and the prime stimulus is CW (CCW percept predicted by FS), which influence dominates? To investigate this, we again fit a binomial GLMM predicting whether the participant's response followed the SI-consistent percept. The model included surround contrast (spatial context), blank duration (temporal context), and their interaction as fixed effects, along with a random intercept for participants.

Model comparisons revealed that both factors contributed significantly to the model fit. Adding temporal context to a spatial-only model significantly improved the fit, χ^2^(3) = 51.76, *p* < 0.001, ΔR²_McFadden_ *=* 0.019, ΔAIC = 45.76, while adding spatial context to a temporal-only model led to a much greater improvement, χ^2^(1) = 481.34, *p* < 0.001, ΔR²_McFadden_ *=* 0.176, ΔAIC = 479.34, suggesting a stronger influence of spatial cues. Adding the interaction term, however, did not significantly improve the fit, χ^2^(3) = 1.53, *p* = 0.676, ΔR²_McFadden_ *=* 0.001, ΔAIC = 4.47, indicating no reliable modulation of one factor by the other.

**Figure 5.**
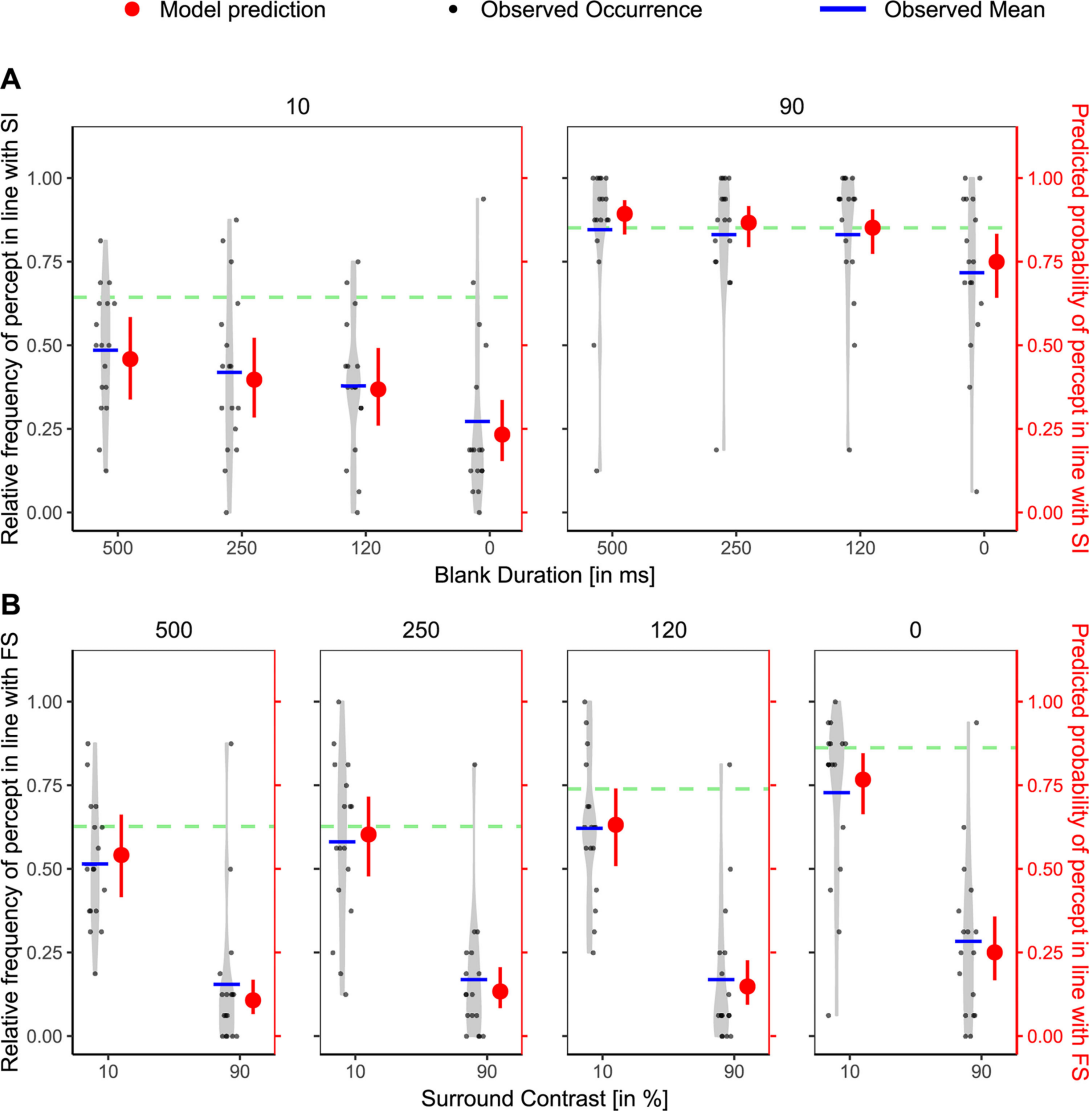
Results from competitive trials in Experiment 1. (**A**) Relative frequency of the percept reported in line with the SI paradigm (black, left *y* axis) per participant and corresponding model predictions (red, right *y* axis) across different levels of the blank duration, with the left depicting low-contrast (10%) and the right high-contrast (90%) trials. Light gray violin plots represent the distribution of individual data, jittered black dots indicate the observed values, and blue horizontal lines show the observed condition means. Red points with error bars show model-predicted means and their 95% CIs. (**B**) Same as (**A**) but with the percept reported in line with the FS paradigm, with different panels for the different blank durations (ms). For reference, the average observed baseline values (SI baseline for A and FS baseline for B) are plotted as a dashed light green line, respectively.

In the best-fitting model (excluding the interaction), the effect of spatial context was robust: the stronger surround (90%) increased SI-consistent responses compared with the reference condition (10% contrast, 500-ms blank duration; β = 2.29, SE = 0.12, z = 19.54, *p* < 0.001). Estimated log-odds for the temporal effects were comparatively smaller (250 ms, β = −0.25, SE = 0.15, z = −1.66, *p* = 0.097, 120 ms, β = −0.37, SE = 0.15, z = −2.48, *p* = 0.013, and 0 ms, β = −1.03, SE = 0.15, z = −6.76, *p* < 0.001), suggesting that shorter blank durations decreased the likelihood of SI-consistent responses, see Figure 5A. Figure 5A also illustrates the lack of interaction effect: for both 10% and 90% contrast surrounds, adding FS results in a moderate decline in the frequency of SI-predicted percepts with decreasing blank duration. The SI-induced strong increase can be seen by comparing the Figure 5A, left and right, for percepts in line with SI and Figure 5B for a visualization of percepts in line with FS.

We selected the different strengths of spatial and temporal influence to be able to make more nuanced statements about which influence was stronger. If we had chosen only one strength for each of the influences and one would have been stronger than the other, it could have been purely because the chosen strength happened to be greater than the other. To more directly compare the relative strength of spatial and temporal effects, we matched their respective baseline effects (Figure 3) using paired Bayesian *t*-tests. Specifically, we compared all combinations of SI baseline and FS baseline trials. We identified three condition combinations where their influence could be considered approximately equal, that is, they biased perception equally strongly in the baseline (Bayes factor in favor of the null hypothesis, BF₀₁ > 3; Jeffreys, 1998): (1) 10% + 500 ms: BF₀₁ = 3.88, (2) 10% + 250 ms: BF₀₁ = 3.85, (3) 90% + 0 ms: BF₀₁ = 3.95, all others BF₀₁ < 1.29.

Using these matched conditions, we then tested the same combinations in competitive trials (i.e., where spatial and temporal contexts influenced perception in opposite directions). Results showed that in the first condition (10% + 500 ms), spatial and temporal context were equal in strength (BF_01_ = 3.83). In the second condition (10% + 250 ms), there was weak evidence for equality (BF_01_ = 1.85). Last, in the third condition (90% + 0 ms), spatial context clearly dominated (Bayes factor in favor of the alternative hypothesis, BF_10_ = 18.94).

Together with the model comparison, these results demonstrate that the spatial context (SI) was more effective in influencing perception than the temporal context (FS) when both were presented in conflict.

#### Clarity ratings: Additive vs. competitive trials

To assess how trial type (additive vs. competitive) influences the subjective clarity of perception, we fit a series of cumulative link mixed models (CLMMs) predicting clarity ratings with random intercepts for participants. Fixed effects included trial type (additive, competing), blank duration, and surround contrast. We began by evaluating each predictor in isolation. Trial type alone significantly improved the model fit over the pure random-intercept model, χ²(1) = 35.09, *p* < 0.001, R²_McFadden_ *=* 0.003, ΔAIC = 33.09, indicating a reliable contribution of trial type to perceived clarity. According to the intercept estimate for additive trials compared with the reference of competitive trials, additive trials had a significantly higher clarity rating than trials in which SI and FS effects were competing, β = 0.34, SE = 0.06, z = 5.91, *p* < 0.001. We then fitted two models including only one of the fixed effects for the other two independent variables (blank duration, contrast) and showed that contrast was by far the strongest single predictor, χ²(1) = 306.82, *p* < 0.001, R²_McFadden_ *=* 0.030, ΔAIC = 304.82, while blank duration showed only a marginal effect, χ²(3) = 6.89, *p* = 0.076, R²_McFadden_ *=* 0.001, ΔAIC = 0.89.

Adding blank duration to the trial type model yielded no substantial improvement, χ²(3) = 7.01, *p* = 0.071, ΔR²_McFadden_ *=* 0.001, ΔAIC = 1.01, but incorporating contrast led to a dramatic increase in model fit, χ²(1) = 308.88, *p* < 0.001, ΔR²_McFadden_ *=* 0.030, ΔAIC = 306.88. Further adding the pairwise interactions still significantly improved the fit, χ²(7) = 45.38, *p* < 0.001, ΔR²_McFadden_ = 0.004, ΔAIC = 31.38, establishing the best-fitting model. Including the three-way interaction significantly improved the fit, χ²(3) = 2.99, *p* = 0.394, ΔR²_McFadden_ *=* 0.000, ΔAIC = 3.01. Thus, in the best-fitting model (the model with all pairwise interactions), the effect of trial type was no longer statistically significant (β = 0.11, SE = 0.13, z = 0.87, *p* = 0.385), suggesting that its influence on trial type may be crowded out by the more substantial effects of the spatial and temporal context variables. However, in simpler models—either with trial type alone or in pairwise combinations—trial type consistently predicted higher clarity ratings for additive over competitive trials. The best-fitting interaction model revealed that clarity is jointly influenced by spatial and temporal context, with trial type slightly modulating these effects (see Supplementary Material S3 for a visualization). These results suggest that clarity is not solely driven by trial structure, but by the specific combinations of spatial and temporal conditions that define that structure.

### Summary

Experiment 1 aimed to investigate the influence of both spatial and temporal contexts on bistable perception by combining both in a BR paradigm. We replicated common findings in surround inhibition and FS. Namely, the FS effect increased with a decreasing blank duration, and the surround exerted a stronger influence on perception (toward a grating dissimilar from the context) when it was high in contrast. Furthermore, we showed that the effect was additive if the predictions of SI and FS were the same in conditions where a single context (temporal or spatial) did not lead to a ceiling effect. We also tested whether the spatial or the temporal context was stronger in disambiguating perception when the predictions of these contexts differed and found a clear-cut preference for spatial cues. Additionally, we found that the clarity ratings were dependent on the level of blank duration, surround contrast, and trial type. Surprisingly, we could not find a general effect of trial type on clarity in the best-fitting model, but only complex interactions.

## Experiment 2

So far, we only examined one temporal parameter, namely, blank duration. This variable is, however, not the only relevant temporal parameter. It has been argued that probe duration also critically influences perceptual dominance in FS (Ikeda et al., 2009; Ikeda & Morotomi, 2007; Wolfe, 1984). In Experiment 2, we set the blank duration to 0 ms to opt for the highest FS effect (because FS was already weaker compared with SI in Experiment 1) and varied the probe duration from short (strongest expected effects based on the literature) (Wolfe, 1984) to long (weakest expected effects) (Wolfe, 1984).

Additionally, some previous research has claimed that blank and probe duration function in the same manner. Specifically, it has been deduced that it does not matter whether the blank or the probe duration changes; rather, their combined time matters (Ikeda et al., 2009; Ikeda & Morotomi, 2007; Wolfe, 1984). Matching our probe durations to the blank durations allowed us to investigate this peculiarity of FS.

The aim of Experiment 2 was twofold. First, we wished to check whether we can replicate the interactive effects of SI and FS already described in Experiment 1. Second, we wanted to examine the influence of the additional FS-temporal parameter: probe duration.

### Methods

#### Participants

As in Experiment 1, a total of 17 participants (14 females and 3 males) participated in Experiment 2. Ages ranged from 20 to 33 years, with an average age of 23.06 ± 3.94 years. The same restrictions and compensations as in Experiment 1 applied.

#### Apparatus and stimuli

The apparatus and stimuli were the same as in Experiment 1.

#### Procedure

The procedure remained largely the same with a few notable exceptions: 1) there was no blank; instead, the probe duration was varied. The duration of the probe was either 120, 250, 500, or 1,000 ms to match the manipulation of the blank duration of Experiments 1, 2) We removed the red fixation dot that had previously indicated that a response needed to be made and instead asked participants to respond when the central stimulus vanished. 3) In Experiment 1, the prime (e.g., CW) and the matching part of the probe (e.g., also CW) were always presented to the same eye (intraocular presentation). The comparison of inter- and intraocular effects (FS has been shown to be stronger in intraocular presentation, where stimulus and eye effects work together) (Ikeda & Morotomi, 2002) will be presented in a future article, together with an additional experiment. Here, we want to focus on the interaction between FS and SI in the intraocular condition and present only data from these trials here. Importantly, the interaction between FS ocularity and probe duration was not significant (Supplementary S4).

Participants completed 16 blocks of 52 trials each, resulting in 832 experimental trials. Completing the experiment took participants up to 60 minutes for each of the two sessions.

### Results

#### Baseline validation

The baseline validation in Experiment 2 served the same dual purpose as in Experiment 1. First, we used the baseline data as reference values when examining additive and competitive trials. Second, we aimed to verify that our implementation of the paradigms in Experiment 2 replicated the general pattern of findings typically observed for FS and SI. Specifically, in the FS baseline, we present a grating (e.g., CW) during the prime and then directly present a probe (e.g., CW to one eye, CCW to the other eye) for varying durations. Here, we anticipated that the preferred stimulus during the probe would generally be the one not previously shown during the prime (i.e., the CCW grating in the example used).

To statistically evaluate whether we could find this general FS effect and the potential influence of probe duration on the perceptual outcome, we again fitted GLMMs with a binomial response (percept in line with FS vs. not in line with FS) and participant as a random intercept.

First, we tested a model with only a random intercept for participant. A Wald test on the intercept revealed that across different probe durations, participants were significantly more likely to perceive the grating not shown during the prime (β = 2.81, SE = 0.28, Z = 9.93, *p* < 0.001, 95% CI 2.25–3.36), showing a general FS effect.

**Figure 6.**
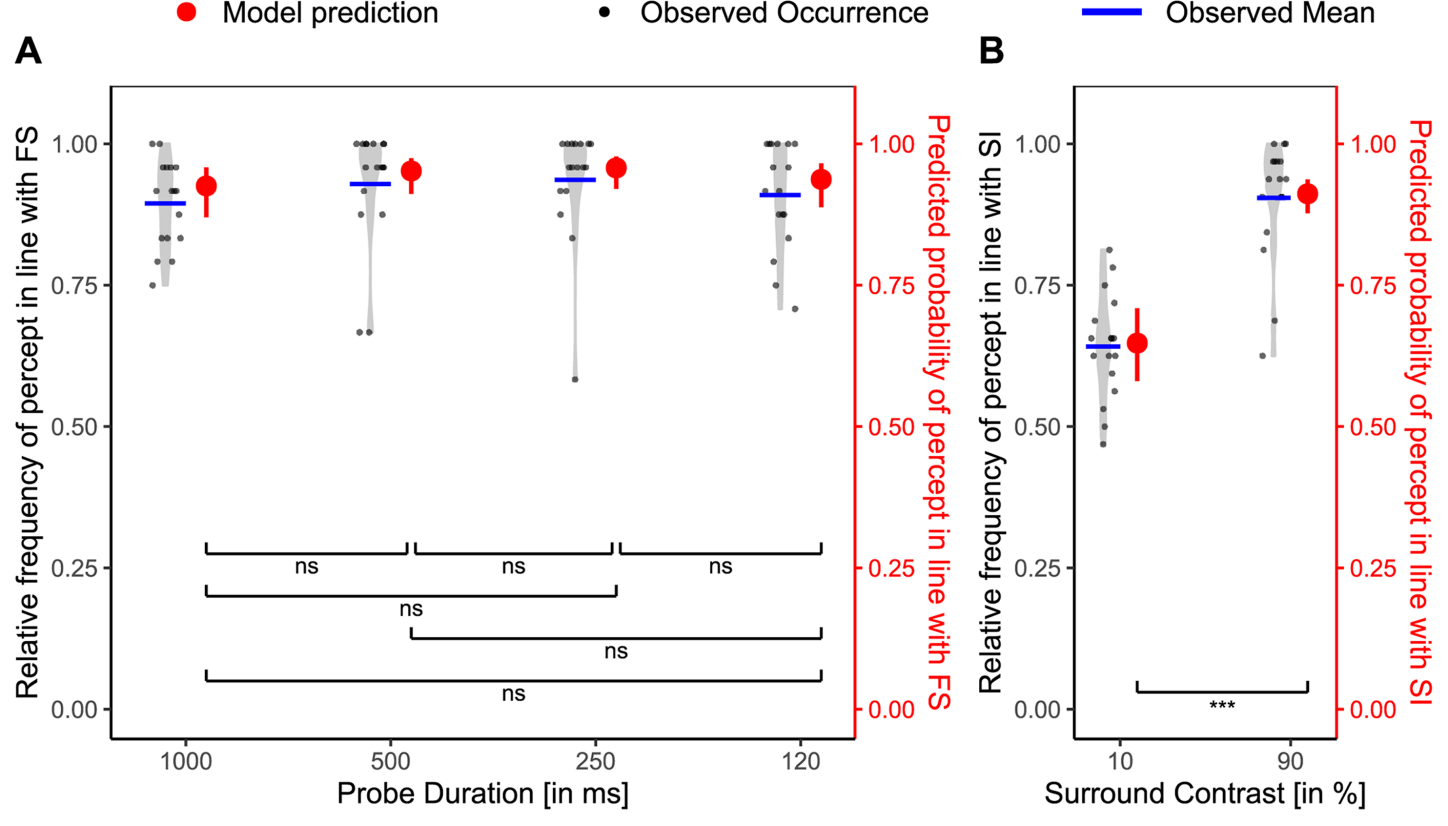
Baseline condition results for FS (**A**) and SI (**B**) in Experiment 2. (**A**) Relative frequency of the percept reported in line with the FS paradigm (black, left *y* axis) per participant and corresponding model predictions (red, right *y* axis) across varying probe durations for the FS baseline condition. Probe durations are ordered on the *x* axis from weakest hypothesized FS effect (500 ms) to strongest (0 ms). Light gray violin plots represent the distribution of individual data, with jittered black dots indicating the observed values, and blue horizontal lines showing the observed condition means. Red points with error bars show model-predicted means and their 95% CIs. Statistical significance is based on Bonferroni-corrected planned comparisons of model predictions. Ns, not significant. *p_adj_ < 0.05; **p_adj_ < 0.01; ***p_adj_ < 0.001. (**B**) Same as (**A**), but for the SI paradigm. Surround contrasts are ordered from weak (10%) to strong (90%) in terms of hypothesized SI strength. All visual conventions from (**A**) apply.

Next, we investigated whether we could replicate previous results (Wolfe, 1984), namely, that a decrease in probe duration would lead to an increase in the FS effect. To assess this effect, we compared the random intercept model with an extended model that included probe duration as a fixed effect. As a reference value, we used the hypothesized weakest condition, that is, with a 1,000-ms probe duration. Surprisingly, the probe duration model did not provide a significantly better fit, LRT: χ^2^(3) = 6.21, *p* = 0.102, R²_McFadden_ *=* 0.007, ΔAIC = 0.21. Thus, although we find a strong FS effect, we could not replicate the effect of probe duration on the participants' perception. As can be seen in Figure 6A, percepts in line with FS were similarly frequent in all probe duration conditions.

Because the SI manipulation was exactly the same as in Experiment 1, we conducted the same analysis. First, we fitted a model that included only the random factor of participant. The resulting intercept indicated that participants perceived the grating different from the surround across contrast levels according to a Wald test (β = 1.27, SE = 0.12, Z = 10.33, *p* < 0.001, 95% CI 1.03–1.51).

To assess the influence of surround contrast on our participants’ perception, we fitted a model including surround contrast. This model significantly improved the fit compared with the random-intercept model, LRT: χ^2^(1) = 116.33, *p* < 0.001, R²_McFadden_ *=* 0.101, ΔAIC = 114.33. For the fixed effect of surround contrast, we found a significant effect for the high-contrast surround (β = 1.73, SE = 0.18, Z = 9.85, *p* < 0.001) compared with the reference level with a low-contrast surround, as shown in Figure 6B. Thus, we could replicate the SI baseline results from Experiment 1 and previous research (Beckmann et al., 2025; Paffen et al., 2006).

#### Spatial and temporal context are (still) additive

Additive effects were calculated as in Experiment 1. Because FS was already at a ceiling in the baseline validation analysis, the additivity analysis based on FS was rendered futile. Hence, we only analyzed the effect of FS on the spatial surround effect. Again, we implemented a model that included only a random intercept for participant. Furthermore, we set up the additivity factor again, this time with five levels: SI baseline, SI baseline + 1,000-ms probe duration, SI baseline + 500-ms probe duration, SI baseline + 250-ms probe duration, and SI baseline + 120-ms probe duration, from weakest to strongest. The model, which included the fixed effect of the additivity factor, improved the fit significantly, LRT: χ^2^(4) = 161.13, *p* < 0.001, R²_McFadden_ = 0.070, and showed a decrease in the information criterion (ΔAIC = 153.13).

**Figure 7.**
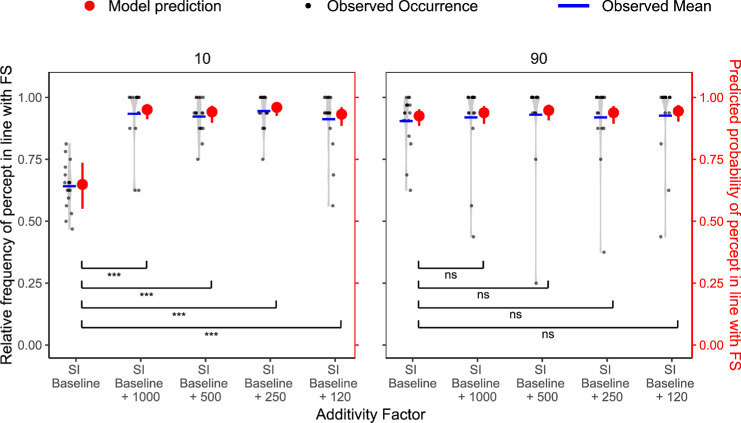
Additivity of FS (modulated by probe duration) and spatial context in Experiment 2. Relative frequency of the percept reported in line with the surround inhibition (SI) paradigm (black, left *y* axis) per participant and corresponding model predictions (red, right *y* axis) across different levels of the additivity factor. The *x* axis is ordered from weakest to strongest, starting with the pure SI baseline to the SI baseline with the weaker FS condition (1,000 ms) down to the strongest FS condition (120 ms). Light gray violin plots represent the distribution of individual data, with jittered black dots indicating the observed values, and blue horizontal lines showing the observed condition means. Red points with error bars show model-predicted means and their 95% CIs. Statistical significance is based on Bonferroni-corrected planned comparisons of model predictions. ns, not significant. *p_adj_ < 0.05; **p_adj_ < 0.01; ***p_adj_ < 0.001.

Next, we fitted a model with surround contrast, which also yielded a significant increase in fit compared with the random intercept model, χ^2^(1) = 59.20, *p* < 0.001, R²_McFadden_ *=* 0.026, ΔAIC = 57.20. A model including both the surround contrast and the additivity factor, provided a better fit compared with the simple additive model, χ^2^(1) = 63.34, *p* < 0.001, ΔR²_McFadden_ *=* 0.028, ΔAIC = 61.34, and also relative to the simple surround contrast model, χ^2^(4) = 165.27, *p* < 0.001, ΔR²_McFadden_ *=* 0.072, ΔAIC = 157.27. Adding the interaction between the additivity factor and surround contrast further increased the model fit, χ^2^(4) = 67.19, *p* < 0.001, ΔR²_McFadden_ *=* 0.029, ΔAIC = 59.19. Similar to previous additivity analyses, the interaction revealed that the influence was especially strong in the weak surround condition compared with the strong, high-contrast condition (Figure 7).

#### Spatial context (still) overrules temporal context

In contrast with the additive trials, we have trials for which we would predict different stimuli to dominate perception, depending on whether the temporal context via FS or the spatial context via SI is more important. To investigate context dominance, we fit a binomial GLMM predicting whether the participant's response followed the SI-consistent percept. The models could include surround contrast and probe duration, as well as their interaction term.

Comparing the models, we found that the model containing surround contrast provided a better fit than the random intercept model, LRT: χ^2^(1) = 619.46, *p* < 0.001, R²_McFadden_ *=* 0.215, ΔAIC = 617.46. Meanwhile, the probe duration did not improve the fit beyond the random intercept model, LRT: χ^2^(3) = 1.86, *p* = 0.601, R²_McFadden_ *=* 0.001, ΔAIC = 4.14. Consequently, adding temporal context to a spatial only model did not improve the fit significantly, χ^2^(3) = 2.56, *p* < 0.464, ΔR²_McFadden_ *=* 0.001, ΔAIC = 3.44, whereas adding spatial context to a temporal-only model led to a better fit, χ^2^(1) = 620.15, *p* < 0.001, ΔR²_McFadden_ *=* 0.215, ΔAIC = 618.15, suggesting a stronger influence of spatial cues. This finding is interesting: in the baseline, there was no modulation by probe duration, possibly owing to a ceiling effect caused by using a 0-ms blank duration (FS is already very strong). Here, however, there was no ceiling effect: the probe duration would have had the possibility to modulate percepts toward FS-predicted percepts (against the SI influence), but it did not. Just as in the previous competitive analysis in Experiment 1, adding the interaction term did not significantly improve the fit, χ^2^(3) = 4.11, *p* = 0.250, ΔR²_McFadden_ *=* 0.001, ΔAIC = 1.89, indicating no reliable modulation of one factor by the other. Thus, the best-fitting model had only one fixed effect—surround contrast—reflecting baseline results; that is, under competitive conditions, predictions were higher for a high-contrast surround compared with a low-contrast surround, β = 2.48, SE = 0.11, z = 22.14, *p* < 0.001.

**Figure 8.**
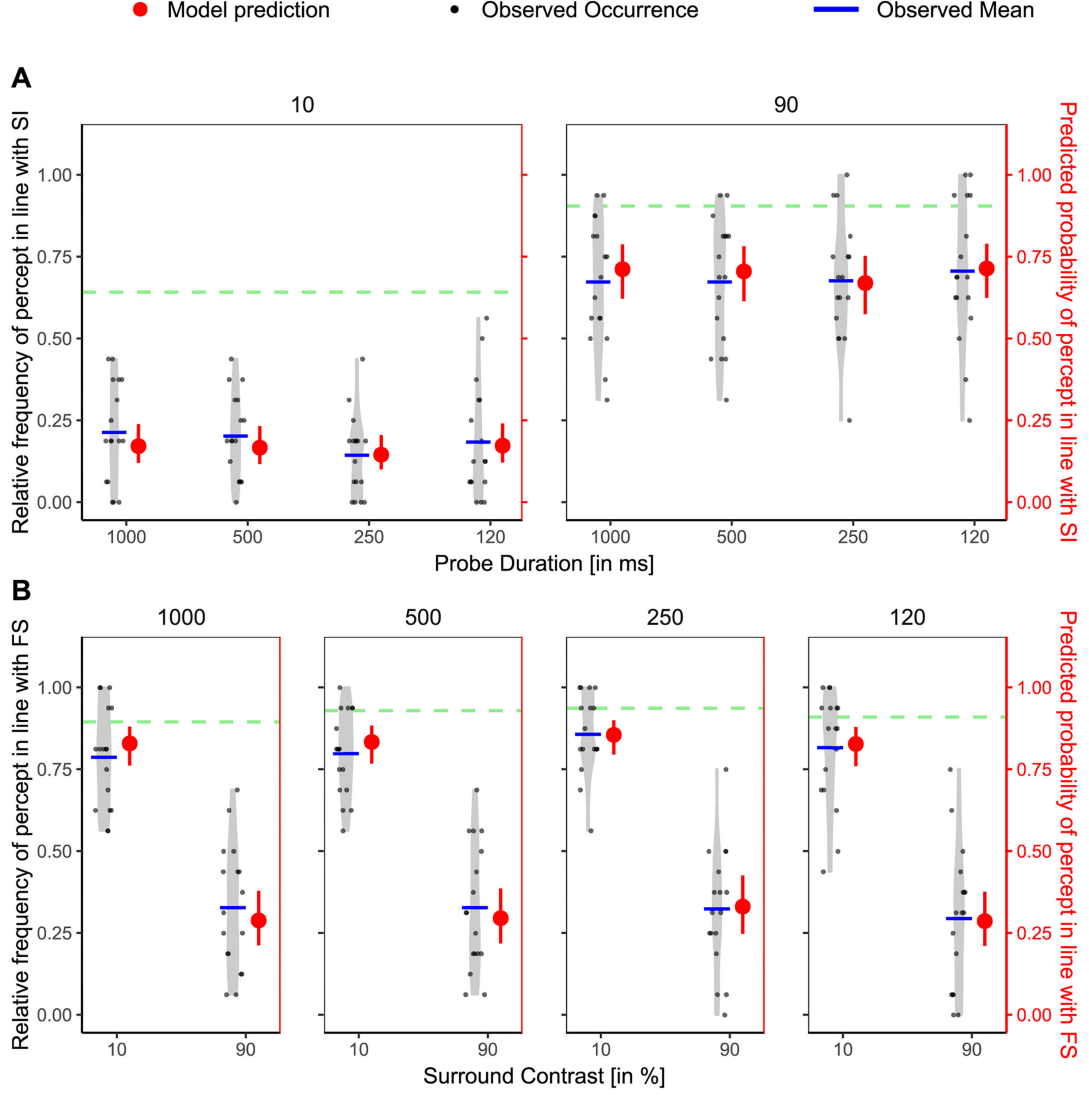
Results from competitive trials in Experiment 2. (**A**) Relative frequency of the percept reported in line with the SI paradigm (black, left *y* axis) per participant and corresponding model predictions (red, right *y* axis) across different levels of the probe duration, with the left depicting low-contrast and the right high-contrast trials. Light gray violin plots represent the distribution of individual data, jittered black dots indicate the observed values, and blue horizontal lines show the observed condition means. Red points with error bars show model-predicted means and their 95% CIs. (**B**) Same as (**A**) but with the percept reported in line with the FS paradigm, with different panels for the different probe durations (ms). For reference, the average observed baseline values (SI baseline for A and FS baseline for B) are plotted as a dashed light green line, respectively.

Taking a closer look at Figure 8A, it appears that we find the same pattern in effect as in Experiment 1, but more pronounced. When the surround is weak (low contrast), temporal context prevails over spatial context, and when the surround is strong (high contrast), spatial context prevails over temporal context. Figure 8B shows the equivalent plot with the *y* axis representing the relative frequency/model prediction of the percept in line with FS.

To compare the strengths of FS and SI directly, we searched for conditions of indistinguishable strength in the baseline and then compared these matching conditions in direct competition. Analogous to the analysis in Experiment 1, we calculated a paired Bayesian *t*-test comparing all combinations of baseline conditions. This helped us identify the three condition combinations that are considered “equal” according to their Bayes factor (BF_01_ > 3): 1) 1,000-ms probe duration and 90% surround contrast: BF_01_ = 3.78, and (2) 120-ms probe duration and 90% surround contrast: BF_01_ = 3.95.

We then tested these matched combinations in the competitive trials by calculating another paired Bayesian *t*-test, comparing the occurrences in line with SI and occurrences in line with FS. Results showed that, in both combinations, spatial context clearly dominated, 100 ms + 90%: BF_10_ = 21.76 and 120 ms + 90%: BF_10_ = 32.16. Overall, our findings in Experiment 2 suggest (just as in Experiment 1) that spatial cues (SI) are more influential than temporal cues (FS) in disambiguating ambivalent stimuli.

#### Clarity ratings: Additive vs. competing trials

Similarly, as in Experiment 1, we investigated how clarity was affected by trial type, surround contrast, and, in this case, probe duration. To assess the effect of these variables, we fit CLMMs predicting the clarity rating. All models with a single fixed effect, provided a significantly better fit than the intercept only model, trial type: χ²(1) = 262.36, *p* < 0.001, R²_McFadden_ *=* 0.027, ΔAIC = 260.36; surround contrast: χ²(1) = 62.76, *p* < 0.001, R²_McFadden_ *=* 0.006, ΔAIC = 60.76; and probe duration: χ²(3) = 213.29, *p* < 0.001, R²_McFadden_ *=* 0.022, ΔAIC = 207.29. Adding probe duration to the trial type model yielded a significant improvement in fit, χ²(3) = 226.80, *p* < 0.001, ΔR²_McFadden_ = 0.023, ΔAIC = 220.80. Further adding surround contrast again improved the fit, χ²(1) = 67.94, *p* < 0.001, ΔR²_McFadden_ *=* 0.007, ΔAIC = 65.93.

**Figure 9.**
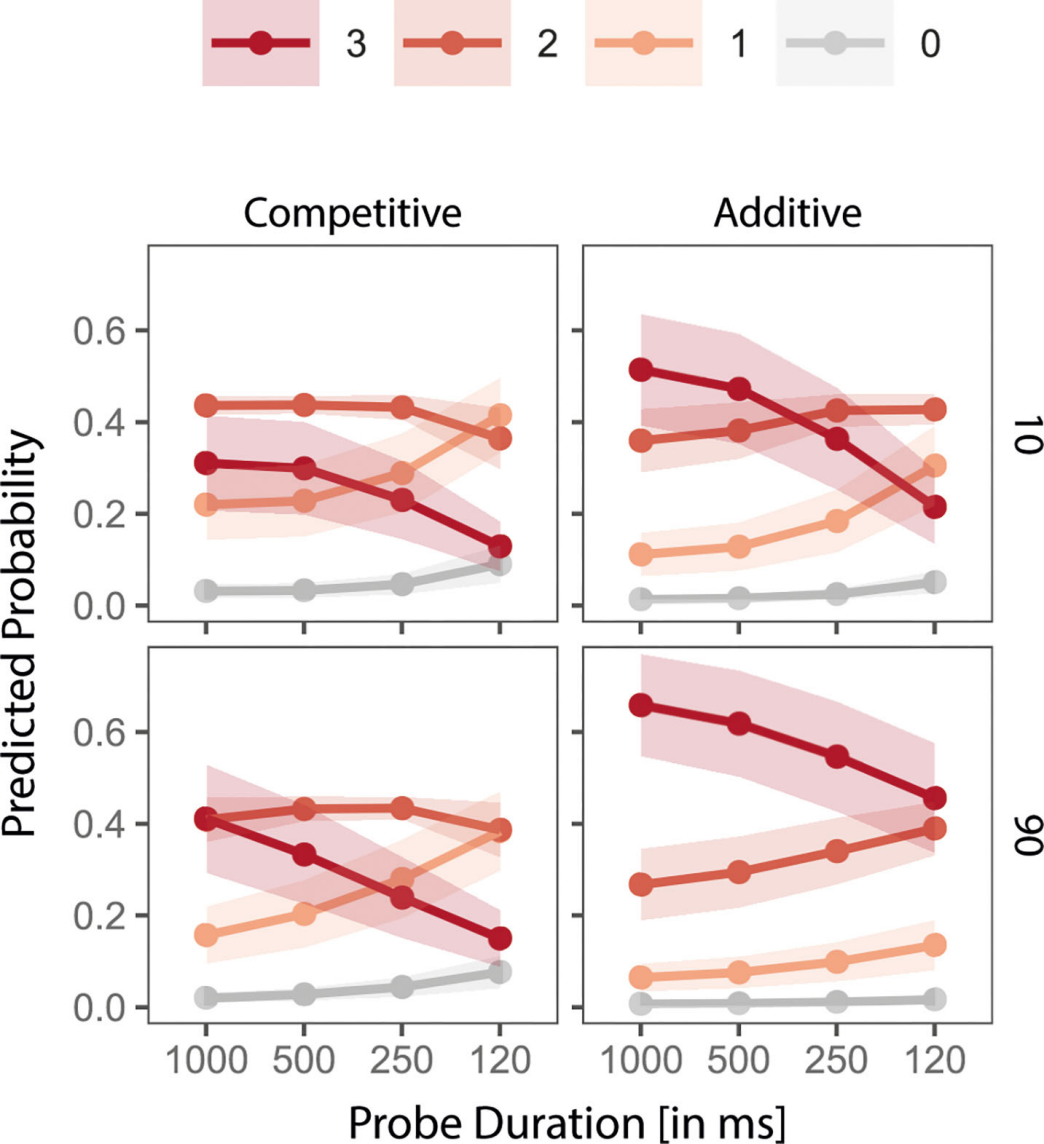
Results from clarity ratings in Experiment 2. Predicted probabilities of clarity ratings (0–3) from a CLMM, as a function of probe duration (*x* axis), surround contrast (in %) (rows), and trial type (columns). Lines represent predicted probabilities for each rating level, with shaded ribbons indicating 95% CIs. Higher values on the *y* axis indicate greater model-predicted likelihood of a given clarity rating. The equivalent plot for the data from Experiment 1 can be found in the Supplementary material for comparison as Supplementary Figure S2.

The best-fitting model included all two-way interactions, χ²(7) = 28.56, *p* < 0.001, ΔR²_McFadden_ *=* 0.003, ΔAIC = 14.56, but not adding the three-way interaction, χ²(3) = 6.10, *p* = 0.107, ΔR²_McFadden_ *=* 0.001, ΔAIC = 0.10. Unlike in Experiment 1, in the best-fitting model including the pairwise interactions, we find a clear-cut effect of trial type: additive trials lead to higher clarity, compared with competitive trials (β = 0.66, SE = 0.13, z = 4.93, *p* < 0.001) (Figure 9). A high surround contrast only led to higher clarity ratings when trials were additive (β = 0.58, SE = 0.12, z = 4.90, *p* < 0.001), but not in competitive trials (β = 0.24, SE = 0.13, z = 1.81, *p* = 0.070). Somewhat surprisingly, clarity decreased with decreasing probe duration in competitive trials (500 ms: β = −0.11, SE = 0.14, z = −0.80, *p* = 0.426, 250 ms: β = −0.53, SE = 0.14, z = −3.72, *p* < 0.001, 120 ms: β = −1.29, SE = 0.14, z = −9.06, *p* < 0.001).

### Summary

Experiment 2 aimed to investigate the influence of the probe duration on FS and the interaction of spatial and temporal context. For pure FS, no effect of probe duration on prediction by FS was shown. Even when FS was competing with the spatial surround and thus not at ceiling, we still did not see any effect of probe duration or an interaction (Figure 8). This result suggests that probe duration is not an effective variable in manipulating the strength of FS.

We could replicate the additivity of the factors SI and FS, at least in a condition that was not affected by ceiling effects (i.e., the low-contrast SI condition(s)). In this respect, Experiment 2 did not provide relevant information that went beyond what was already found in Experiment 1.

Interestingly, despite using the strongest possible settings for the FS task (blank 0 ms with 120-ms probe duration), we again found that, when spatial cues were added (in the form of [high-contrast] SI), those spatial cues determined the perceptual outcome. Regarding clarity, Experiment 2 provided much clearer results than those of Experiment 1. Clarity was higher in additive trials than in competitive trials. This effect interacted with surround contrast: only in additive trials did the higher surround contrast improve clarity compared with the lower surround contrast. Furthermore, we found that clarity ratings seemed to increase as the probe duration increased, which is counterintuitive given that, according to the literature, FS should get stronger with decreasing probe duration. With stronger effects, clear percepts would be expected. Instead, we could not replicate the modulation of FS strength by probe duration and found that percepts actually became clearer with increasing probe duration.

## Between experiment comparison (exploratory data analyses)

### Perceptual clarity ratings: Are they comparable with 2AFC results?

Previously, authors have used ratings as answer types in FS paradigms. For example, Wolfe's (1984) participants answered 5 if the stimulus appeared entirely horizontal, −5 if it appeared entirely vertical, and 0 if observers could not decide between the two. He is not the only researcher who has used these types of ratings (Supplementary Table S1). In Wolfe's paper, results based on ratings did not differ from results based on a forced choice paradigm (Wolfe, 1984).

In addition to the 2AFC response acquisition, we also used clarity ratings as a further measurement. Therefore, we wanted to take the opportunity to see what could be learned. Prior literature primarily emphasizes the comparison of these two methods within the FS framework (e.g., Wolfe, 1984), which is why we focus here on comparing them in the FS conditions without SI manipulation, that is, the FS baseline.

**Figure 10.**
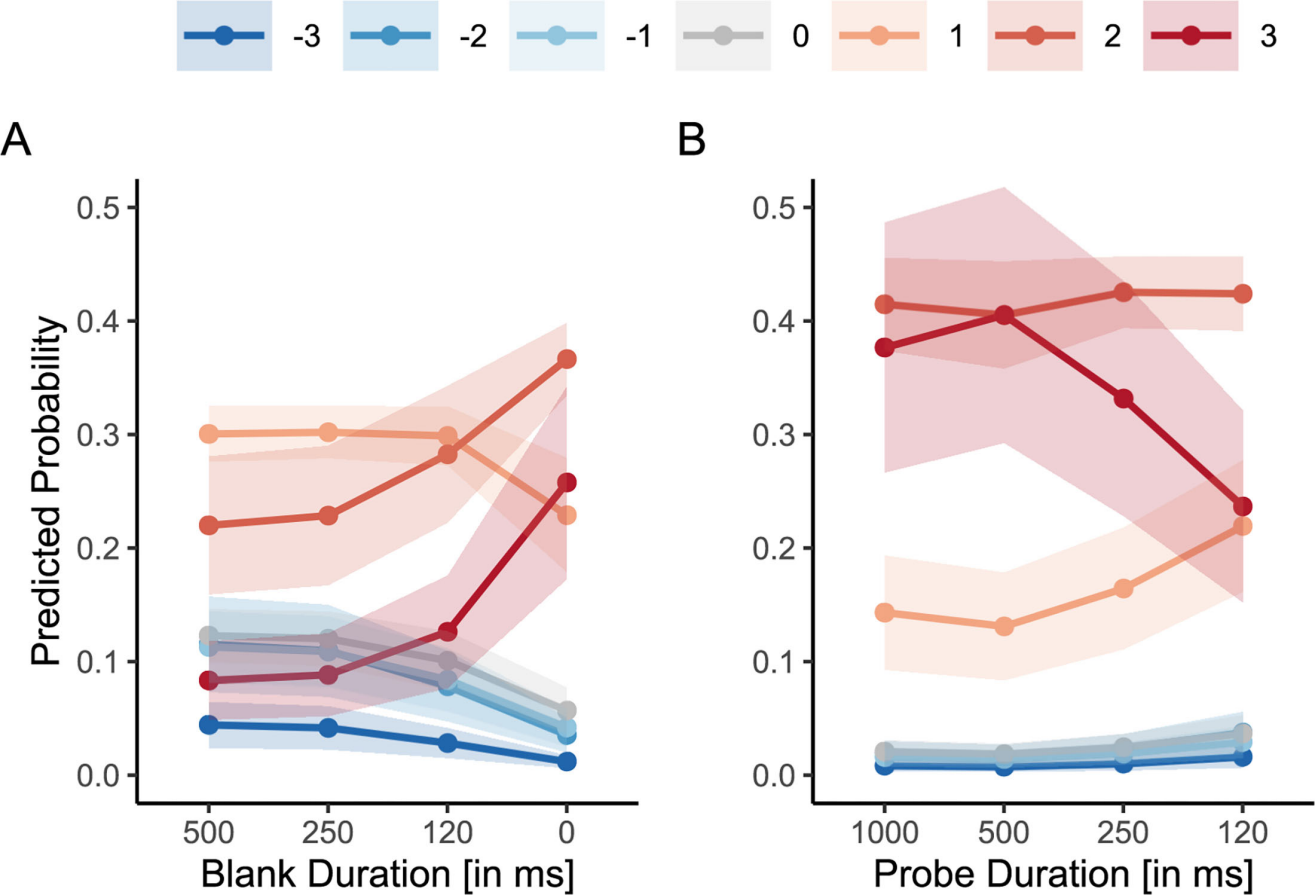
Transformed clarity ratings in the FS baseline in Experiment 1 (**A**) and Experiment 2 (**B**). Transformed ratings reflect alignment with FS predictions, where positive values indicate perceptual dominance in line with expected FS effects, and negative values indicate the opposite. Random intercepts were modeled for participant ID in both analyses. (**A**) Predicted probabilities from a CLMM with blank duration as a fixed effect. The *x* axis represents blank duration (500, 250, 120, and 0 ms), and the *y* axis shows the predicted probability of each transformed clarity rating (ranging from −3 to +3), with shaded ribbons indicating 95% CIs. (**B**) Predicted probabilities from a separate CLMM including probe duration (1,000, 500, 250, and 120 ms) as a fixed effect.

Thus far, we looked at the clarity rating as an additional measure to characterize the subjective, phenomenological experience of the observers. However, now we wish to use our clarity ratings to examine to what extent 2AFC measures and clarity ratings produce comparable results and thus to check whether it is justifiable to interchange those measures in studies on BR. To make our clarity rating comparable to that used in the previous literature (e.g., from −5 if participants clearly saw one stimulus to +5 if they clearly saw the other) (Wolfe, 1984), we used the following transformation: When the response was in line with what FS would predict, the rating remained as it was. If the response was opposite to the FS prediction, we applied a sign reversal by multiplying it by −1 to obtain a negative value. We refer to the resulting variable as the transformed rating (e.g., see Figure 10).

Figure 10A shows that the clarity of the percepts is in line with FS increased with decreasing blank duration. This observation could also be demonstrated statistically: we fitted two CLMMs—one random intercept model and one model including blank duration as a fixed effect predicting the transformed clarity rating. The model including blank duration provided a much better fit, LRT: χ²(3) = 187.79, *p* < 0.001, R²_McFadden_ *=* 0.025, ΔAIC = 181.79. Pairwise comparisons were conducted using estimated marginal means, adjusted for multiple comparisons using the Bonferroni method. Except for the difference between 500 and 250 ms, all pairwise differences were significant.

Interestingly, Figure 10B, depicting the results from Experiment 2 on the effect of probe duration, tells a different story: Clarity of percepts predicted by FS decreased as the probe duration decreased, marking a dissociation between ratings and 2AFC (cf., Figure 6B). We again fitted two CLMMs—an intercept-only model and a model additionally including probe duration as a fixed effect. Unlike in the analysis for the 2AFC data, adding probe duration as a factor to the model significantly increased the model fit, LRT: χ²(3) = 42.10, *p* < 0.001, R²_McFadden_ *=* 0.010, ΔAIC = 36.10. Furthermore, many of the post hoc comparisons also became significant (Table 1).

**Table 1.** Post hoc comparisons of model-predicted transformed clarity ratings; results from different probe durations were compared.

Contrast	β estimate	SE	95% CI	z Value	*p*_adj_ Value
1,000 > 500	0.12	0.14	[−0.24 to 0.48]	0.89	1.000
1,000 > 250	−0.20	0.13	[−0.55 to 0.16]	−1.47	0.843
1,000 > 120	−0.67	0.13	[−1.02 to −0.32]	−5.02	<0.001
500 > 250	−0.32	0.13	[−0.67 to 0.03]	−2.40	0.099
500 > 120	−0.79	0.13	[−1.14 to −0.44]	−5.98	<0.001
250 > 120	−0.47	0.13	[−0.81 to −0.13]	−3.64	0.002

*Notes*: CI, confidence interval; SE, standard error.

Thus, we found that the transformed rating decreased when the probe duration decreased, but increased as the blank duration decreased. Taken together, these results suggest differences in the rating results between blank and probe durations. In the next section, we discuss the distinction between the effects of blank and probe duration.

### Blank duration and probe duration do not function in the same manner

The last analysis showed that the probe and blank duration did not work in the same manner. Increases in blank duration decreased the suppressive effect of the prime, but increases in probe duration had little effect on prime-induced suppression. Thus, our findings are in contrast to what previous studies reported. Previously, it was found that increases in probe duration had the same effect as increases in the blank duration. Wolfe (1984) states about the probe duration:

For test flashes longer than 200 ms [= probe duration], the degree or frequency of suppression declines just as it declines when the ISI [= blank duration] becomes longer than 200 ms. Flashes a full second long [= probe duration] are suppressed about as effectively as are 10 ms flashes that occur 1000 ms [= blank duration] after the offset of the initial variable. (p. 476)

In fact, it was argued that the two variables, blank duration, and probe duration, could be combined into one variable (e.g., Ikeda et al., 2009; Ikeda & Morotomi, 2007; Wolfe, 1984). The group around Ikeda called this SOFFA.

However, we used different durations in our experiment that could explain this discrepancy. Luckily, there were two conditions in the two experiments, respectively, in which we could more directly test whether the probe and blank duration acted similarly since they amounted to the same SOFFA: Condition 1 is from Experiment 1. Here, the probe duration was 250 ms, and blank duration was 250 ms (SOFFA: 500 ms). Condition 2 is from Experiment 2 (probe duration: 500 ms, blank duration: 0 ms, SOFFA: 500 ms). The SOFFA was thus the same, with differing blank and probe durations.

Contrary to predictions by Ikeda et al. (2000), the two conditions yielded different perceptual outcomes. In condition 1 (blank 250 ms/probe 250 ms), FS was only moderately effective: 62.68% as predicted by FS (median = 65.63%, standard deviation = 19.21). In contrast, in condition 2 (blank 0 ms/probe 500 ms), FS was much stronger: 92.89% (median = 95.83%, standard deviation = 10.70). To statistically test these differences, we combined the data from the FS baseline with a 250-ms blank duration in Experiment 1 and from the FS baseline with a 500-ms probe duration in Experiment 2. We calculated two GLMMs—a random intercept model and a model including time structure (levels 250 ms + 250 ms, 0 ms + 500 ms) as a fixed effect. The second model, including the time structure, provided a better fit, χ²(1) = 25.14, *p* < 0.001, R²_McFadden_ *=* 0.028, ΔAIC = 23.14. Importantly, the 0 ms + 500 ms revealed a higher prediction of percepts in line with FS compared with the 250 ms + 250 ms, β = 2.47, SE = 0.45, z = 5.45, *p* < 0.001, indicating (in conjunction with our findings about the clarity ratings, see Section 4.1) that blank and probe duration did not work in the same way.

## General discussion

Both spatial and temporal contexts are known to affect bistable perception. However, little is known about the interaction between spatial and temporal contexts. To investigate spatiotemporal interactions in the form of context effects in BR, we implemented a novel paradigm combining both context types. The spatial context was provided in the form of a surrounding annulus that could match one of the stimuli of the rivalry target, producing dominance of the percept not matched to the surround, a phenomenon known as surround inhibition. In contrast, the temporal context was implemented via a FS paradigm, producing dominance of the grating not shown during the prime presentation. We varied the strength of the spatial context with a variation of contrast (Experiments 1, 2). Meanwhile, the strength of the temporal context was manipulated by varying the blank duration (Experiment 1) or the probe duration (Experiment 2). We sought to investigate the additivity of spatial and temporal context and which one would exert the stronger influence when the two types of context were put into competition.

In the first experiment, we showed that the number of trials with percepts that were predicted by FS decreased as the blank duration increased. However, we could not find the typical decrease with increasing probe duration in the second experiment. In both experiments, the influence of the surround (SI) on the final perceptual outcome increased with the contrast of the surround.

Spatial and temporal contexts worked additively when both predicted the same percept. Whether additivity could be found depended on whether the baseline condition would allow room for improvement. When contexts were competing, the spatial context exerted a stronger influence than the temporal context.

In both experiments, transformed clarity ratings were higher for the additive condition combination than for competing condition combinations. In Experiment 1, the rating reflected the pattern observed based on the 2AFC format for varying blank durations. Interestingly, a different pattern emerged in Experiment 2 when comparing 2AFC responses for varying probe durations (i.e., no effect) to the rating data (i.e., a modulation by probe duration: increased clarity of responses in line with FS with increasing probe duration).

A direct comparison of the same SOFFA, a combination of blank and probe duration (here: 500 ms), showed that they did not function in the same manner. Specifically, we found that FS was much stronger when the probe duration was 500 ms and the blank duration was kept at 0 ms, compared with when the probe and blank duration were both 250 ms long. Furthermore, the blank duration strongly modulated the participants’ percepts (Experiment 1), while the probe duration had relatively little influence on perception (Experiment 2).

### Spatial and temporal context work additively

The results from Experiments 1 and 2 indicate an additive effect in bistable perception. When one grating was weakened—such as through suppression by FS—it could be further weakened by a surround effect, provided that ceiling effects were not a limiting factor. Even at ceiling, additive processes were likely still at play, although they may not have been detectable in our behavioral data. This stacking of effects within BR adds to previous findings: When increasing the size of a surrounding stimulus, its influence on the perception of a BR center increases (Beckmann et al., 2025; Xing & Heeger, 2001). We can extend this finding by showing that there is not only a summation of spatial influences but also across modulations, that is, temporal and spatial.

### Spatial context overrules temporal context in competition

The dominance of spatial context over the temporal context was demonstrated with strong evidence in both Experiments 1 and 2. Although it was not the case that spatial context won over temporal context no matter what (i.e., the low-contrast surround was not stronger than a strong FS condition), evidence taken from baseline matching strongly argues for a greater impact of the spatial context. There are some reasons why spatial context might be more informative when disambiguating rivalry targets. SI is assumed to rely on both low-level, that is, lateral inhibition (Pack, Hunter, & Born, 2005) and lateral facilitatory connections (Sillito et al., 1995), as well as high-level processing of configurations for object detection: For disambiguation, the brain often relies on different Gestalt principles, emphasizing the importance of spatial relationships and configurations in perceptions to organize the visual input into a cohesive and meaningful pattern based on spatial arrangements (Wagemans et al., 2012). Specifically, in the case of center-surround interactions, the underlying mechanism seems to be a figure–ground segmentation (Born, Groh, Zhao, & Lukasewycz, 2000). Figure–ground segmentation can be interpreted as a marker for processes higher up in the visual processing stream and not just as a consequence of low-level effects, as demonstrated by high-level semantic filling (Muckli et al., 2015; Smith & Muckli, 2010) and the fact that familiarity with a configuration influences the initial determination of figure and ground (Gibson & Peterson, 1994; Vecera & Farah, 1997). The spatial importance might further be strengthened due to the context being presented at the same time as the disambiguating stimulus. Functional magnetic resonance imaging studies have shown that, when multiple stimuli appear simultaneously, they are processed together rather than independently, engaging in mutual suppression and competing for neural representation within the visual cortex (Beck & Kastner, 2005). The temporally distinguishable stimulus might not have the same benefit.

The neural mechanisms underlying bistable perception are closely tied to the activity of specific brain areas within the visual cortex. Notably, many of these areas are also involved in encoding spatial relationships and configurations, suggesting that the competition between alternative perceptual interpretations is partly rooted in the brain's mechanisms for spatial processing (De Valois, Albrecht, & Thorell, 1982; Maffei & Fiorentini, 1973; Press, Brewer, Dougherty, Wade, & Wandell, 2001).

On the other side, the FS effect is mainly assumed to be a low-level effect (i.e., dependency on retinal location, eye-of-origin, and stimulus phase) (Brascamp et al., 2007), partly driven by neuronal adaptation to the prime stimulus. This is further supported by neurophysiological studies with macaques, which suggest FS occurs locally in V1, even in the absence of top-down feedback due to anesthesia (Bahmani, Murayama, Logothetis, & Keliris, 2014).

Spatial information contributes to the overall organization and interpretation of the stimulus, guiding the brain in selecting one perceptual interpretation over the other. Thus, in the present study, we have the spatial context, which is strong both in high and low levels of the perceptual processing hierarchy, competing with FS, mostly known to rely on low-level effects. These factors (Gestalt principles and the combination of bottom-up plus top-down effects of spatial cues) might explain why spatial cues tend to dominate the final percept in the presence of spatial and temporal cues.

Of course, the current experimental paradigm investigates just two instances of many possible experimental manipulations of spatial and temporal contexts, and it will be interesting to examine whether different modulations yield similar results.

### Clarity ratings provide further insights into bistable perception

With 2AFC queries, we force participants to make a decision about their perception in BR. However, bistable perception is seldom a zero-sum game, in which one stimulus, and one stimulus only, is perceived when a bistable stimulus is presented for a longer time. Rather, bistable perception often leads to different levels of uncertainty. To give value to this uncertainty, we introduced our clarity ratings. Generally, we found clarity ratings to be higher in additive context constellations when compared with competitive context constellations.

Interestingly, the clarity ratings did not simply mirror the 2AFC data. Although they closely followed the 2AFC pattern for blank duration, this was not the case for variations in probe duration. Specifically, when using the relative frequency of percepts in line with FS as the dependent variable, there was no room for improvement, which was reflected in the stability of the relative frequency as probe duration increased. In contrast, when context conditions were in competition, predictions should, in principle, have had room to increase with longer probe durations, yet they did not (Figure 8). However, when considering clarity ratings in competitive trials, we observed an increase in ratings with increasing probe durations (Figure 9). The fact that ratings increased with a prolonged probe duration makes sense in terms of what is known about BR: rivalry takes time to resolve (e.g., Bartels & Logothetis, 2010), and thus, the percept should be clearer with prolonged probe duration. These divergent results between 2AFC and clarity ratings suggest that, although the frequency of FS-consistent percepts did not increase, participants’ perceptual decisions became more certain. Thus, clarity ratings serve as a valuable complement to 2AFC measures, providing additional insight into perceptual confidence.

### Differentiating between blank and probe durations in FS: Why combining them as SOFFA might conceal differences

In the last part of the between-experiment comparison, we found that blank and probe durations did not act in the same manner. In our study, we found a ceiling effect that led to the absence of any modulation during the probe duration. However, we found an effect of probe duration on the transformed clarity ratings. More specifically, in the FS baseline, the transformed ratings increased with increasing probe duration but decreased with increasing blank duration. When comparing different combinations of blank and probe duration, which results in the same total duration (blank: 250 ms, probe: 250 ms vs. blank: 0 ms, probe: 500 ms), we found vastly different results: the relative frequency of the grating in line with FS was much higher (65.63% vs. 92.89%) for a short blank duration with a long probe duration compared with when they were the same. This finding challenges the assumption that these two durations can simply be exchanged for one another and that a combination of the two variables into one (SOFFA) is useful (Ikeda et al., 2009; Wolfe, 1984).

Looking at this stark difference between blank and probe duration is interesting in terms of the neurophysiological explanation for FS. The current dominant explanation of FS is neuronal adaptation (e.g., Brascamp et al., 2007; Ikeda & Morotomi, 2002). Adaptation predicts that long prime durations induce adaptation and thereby weaken that stimulus in the subsequent probe presentation. In contrast, the short prime duration will have the opposite effect (flash facilitation; see Brascamp et al., 2007). If a blank is interspersed between prime and probe, the length of the blank interval will determine the extent of recovery from adaptation. Longer blank duration spells better recovery and, thus, less FS. This effect of blank duration could be reliably found in our study.

A closer look at Ikeda et al.’s (2009) parameter choice helps clarify how their results aligned with the idea of the SOFFA, and why ours might diverge. In their study, the prime duration was held constant at 1,000 ms, similar to our experiments, but there were key differences in the configuration and range of blank and probe durations. Most critically, in the experiments from which Ikeda et al. drew their conclusions on the equality of blank and probe manipulation (i.e., the SOFFA), their Experiments 3 and 4, probe durations were held constant or limited to a narrow range (mostly 10 ms), and blank durations were either absent (0 ms) or relatively short. The fact that the relevant temporal parameters were drawn from a very limited range of values may have minimized the differential effects of blank and probe durations, allowing total stimulus duration (SOFFA) to emerge as a more consistent predictor of perceptual outcome. In contrast, our experiments tested longer and more widely spaced blank and probe durations (e.g., 120–1,000 ms for probes), which increased the sensitivity of our experiment to identify the differential contributions of each component. This revealed that longer blank durations allowed more recovery from adaptation and thereby reduced suppression, while longer probe durations did not significantly modulate FS in the 2AFC results. In the transformed clarity ratings, the two variables (blank vs. probe duration) even pushed the transformed clarity ratings into different directions, that is, decreasing clarity with decreasing probe duration and increasing clarity with decreasing blank duration. In essence, Ikeda et al.’s (2009) findings may reflect conditions under which blank and probe durations can be more readily collapsed into a single SOFFA measure, particularly when their individual contribution was relatively muted due to shorter time windows. In contrast, our parameter choices exposed that these durations are not interchangeable, suggesting that SOFFA may obscure important differences in the temporal dynamics in FS when testing a wider range of durations.

Assuming the adaptation account, it seems reasonable that probe duration only has a small effect on FS. In contrast, prime and blank duration play a significant role in neuronal adaptation as they modulate the adaptation and subsequent recovery period. This is what we found with our 2AFC measure for probe duration. In short, our findings are more in line with predictions from the prevailing neurophysiological account of FS and thereby further validate the adaptation explanation (e.g., Brascamp et al., 2007; Ikeda & Morotomi, 2002).

## Conclusions

Our study delved into the interplay between spatial and temporal contexts in bistable perception, aiming to explore their additivity and identify which had priority in competitive scenarios. We found that spatial (SI) and temporal (FS) factors act additively when their respective influences align in the same direction. However, when in competition, spatial factors tend to dominate. Furthermore, contrary to earlier results, probe and blank durations in FS did not exert the same effects in our experiment, indicating that the durations of these two intervals cannot be arbitrarily exchanged or merged. In particular, we found that probe duration had a negligible effect, further supporting the idea that FS is based on neural adaptation.

Additionally, we found that adding a rating to a 2AFC task could uncover otherwise invisible effects. Our findings suggest that perceptual quality partially reflects the consistency of sensory evidence. In conclusion, this study marks an initial effort to investigate, compare, and characterize the interaction of various factors involved in the disambiguation of bistable perception, hoping to inspire future studies using further operationalizations and factors.

## Supplementary Material

Supplement 1
