# Supplementary material for: When contexts collide: Spatial context prevails over temporal context in binocular rivalry
Source: J Vis. 2025 Sep 16;25(11):11. doi: 10.1167/jov.25.11.11 (PMC12448142; doi:10.1167/jov.25.11.11)
Supplement: Supplement 1 [file jovi-25-11-11_s001.pdf]

S. Supplement

S.1 Literature Overview of Flash Suppression

Table S1

Citation	Priority	Exp.	Sample	Prime Duration Min	Prime Duration Max	Blank Duration Min	Blank Duration Max	Probe Duration Min	Probe Duration Max	Intraocular / Interocular / Binocular	Query
Brascamp et al. (2007)	high	1	7	12	2500	71	412	235	235	Intra/Inter/Bino	2AFC
Holmes et al. (2006)	high	1	3	1000	1000	0	0	1000	1000	Bino	3AFC
Holmes et al. (2006)	high	2	3	1000	1000	0	0	1000	1000	Bino	3AFC
Holmes et al. (2006)	high	3	3	1000	1000	0	0	1000	1000	Bino	3AFC
Ikeda & Morotomi (2000)	high	1	4	1000	1000	0	0	10	200	Intra	3AFC
Ikeda & Morotomi (2000)	high	2	4	1000	1000	0	0	10	200	Bino	rating
Ikeda & Morotomi (2000)	high	3	4	1000	1000	10	200	10	10	Bino	rating
Ikeda & Morotomi (2002)	high	1	4	1000	1000	0	0	200	200	Bino	3AFC
Ikeda & Morotomi (2002)	high	2	4	1000	1000	0	0	200	200	Intra/Inter	3AFC
Ikeda & Morotomi (2007)	high	1	4	1000	1000	0	0	10	200	Intra	2AFC
Ikeda & Morotomi (2007)	high	2	4	1000	1000	0	0	10	200	Intra	2AFC
Ikeda et al. (2009)	high	1	4	1000	1000	10	200	10	200	Intra	3AFC
Ikeda et al. (2009)	high	2	4	1000	1000	0	0	10	200	Bino	3AFC
Ikeda et al. (2009)	high	3	4	1000	1000	0	0	10	200	Bino	rating
Ikeda et al. (2009)	high	4	4	1000	1000	10	200	10	10	Bino	rating
Ikeda et al. (2009)	high	5	4	1000	1000	0	2000	10	200	Intra	rating
Ooi & Loop (1994)	high	1	4	50	50	0	0	20	20	Intra	2AFC*
Tsuchiya et al. (2006)	high	2	3	500	500	0	0	Resp*	Resp*	Intra	2AFC
Wolfe (1984)	high	1	5	1000	1000	0	2000	10	10	Intra	rating
Wolfe (1984)	high	2	7	1000	1000	0	1000	10	10	Intra	2AFC
Wolfe (1984)	high	3	10	10	1000	0	2000	10	10	Intra	rating
Wolfe (1984)	high	4	4	2000	2000	10	5000	10	2000	Intra	rating
Bartels & Logothetis (2010)	medium	1	6	500	500	0	0	300	3000	Intra	2AFC
Brascamp & Blake (2012)	medium	1	7	2000	2000	0	0	2000	2000	Intra	rating
Kreiman et al. (2002)	medium	1	14	1000	1500	0	0	300	500	Intra	2AFC
Ling & Blake (2009)	medium	1	4	2500	2500	100	100	1000	1000	Intra	2AFC
Ling & Blake (2009)	medium	2	3	2500	2500	100	100	1000	1000	Intra	2AFC
Meng et al. (2007)	medium	2	4	5000	6000	0	0	Resp	Resp	Intra	2AFC
Meng et al. (2007)	medium	3	12	2000	3000	0	0	Resp	Resp	Intra	2AFC
Moradi & Heeger (2009)	medium	6	6	500	500	0	0	1500	2000	Bino	neuronal

Nichols & Wilson (2009)	medium	1	4	4000	4000	0	0	500	500	Intra/Bino	rating
Parker & Alais (2007)	medium	4	4	375	2750	0	0	500	500	Intra	2AFC
Pearson & Clifford (2005a)	medium	1	4	750	750	3000	3000	750	750	Bino	2AFC
Pearson & Clifford (2005a)	medium	2	4	750	750	3000	3000	750	750	Bino	2AFC
Pearson & Clifford (2005a)	medium	3	4	750	750	3000	3000	750	750	Bino	2AFC
van Ee (2011)	medium	1	8	2500	2500	0	0	500	5000	Intra	detection
Bahmani et al. (2014)	low	1	4M	2000	2000	0	0	2000	2000	Intra	neuronal
Bahmani et al. (2014)	low	2	4M	1000	1000	0	0	1000	1000	Intra	neuronal
Bahmani et al. (2019)	low	1	2M	1000	1000	0	0	1000	1000	Bino	neuronal
Bahmani et al. (2019)	low	2	2M	1000	1000	0	0	1000	1000	Bino	neuronal
Gilroy & Blake (2005)	low	3	4	500	500	0	0	4000	4000	Intra	rating
Kanai et al. (2010)	low	3	6	1470	1470	0	0	Resp	Resp	Intra	detection
Kapoor et al. (2018)	low	1	3M	1000	1000	0	0	Resp	Resp	Intra	neuronal
Kapoor et al. (2022)	low	1	3M	2000	2000	0	0	6000	6000	Intra	OKN
Keliris et al. (2010)	low	1	2M	1000	1000	0	0	1000	1000	Intra/Bino	neuronal
Maier et al. (2007)	low	1	2M	850	850	0	0	800	800	Intra	neuronal
Palmer & Ramsey (2012)	low	1	8	3200	3200	110	110	70	70	N/S	2AFC
Palmer & Ramsey (2012)	low	2	10	3200	3200	110	110	70	70	N/S	2AFC
Palmer & Ramsey (2012)	low	3	7	3200	3200	110	500	70	70	N/S	2AFC
Panagiotaropoulos et al. (2012)	low	1	2M	1000	1000	0	0	1000	1000	Intra	neuronal
Pearson & Clifford (2005b)	low	1	4	500	500	3000	3000	3000	3000	Intra	2AFC
Pearson & Clifford (2005b)	low	2	4	500	500	3000	3000	3000	3000	Intra	2AFC
Pearson & Clifford (2005b)	low	3	4	500	500	3000	3000	3000	3000	Intra	2AFC
Sheinberg & Logothetis (1997)	low	2	2M	1000	2000	0	0	Resp	Resp	Intra	2AFC

Literature review of flash suppression considering all peer-reviewed research papers found before 2023. Priority is defined as how relevant we considered the paper for the current study. Under the “high priority” category, all papers that used simple flash suppression experiments whose variations mostly consisted of changes in any of the given durations. “Medium priority” are studies including variations that made it difficult to conclude anything on the durations suitable for our study, e.g., binocular rivalry with integrated blanks. Under “low priority”, we sorted all animal studies or studies with big variations, such as the usage of afterimages, and studies that did not use 2AFC or ratings as their dependent variable. For the sake of clarity, “Exp.” lists the experimental number within a given publication. ”Sample” gives the sample size. If the number is followed by an "M", this means that the sample consisted of macaques. The next columns reveal information on minimum and maximum durations used in the experiment. The next column gives information on whether the prime grating was shown in the same eye (Intra), the other eye (Inter), or both eyes (Bino) during the probe duration. The last column gives information on how participants were asked for what they saw, this was mostly 2AFC between two percepts, 3AFC between two percepts, and a mixed percept or a rating with the ends representing one of the two percepts. In some cases, detection rate, neuronal response, or optokinetic nystagmus (OKN) were used to measure detections. However, these studies are not particularly relevant for the current study. An asterisk (*) marks when information was not explicitly stated and is our best guess in understanding the study. N/S refers to when information was not specified.

S.2 Additional Additivity Analyses (Exp. 1)

For Exp. 1, we reported the analysis of additive effects from spatial factors (SI) on temporal (FS), when both push the same percept during the probe. Here, we report the mirror analysis, which investigated how FS would enhance the SI predictions, yielding similar results to the effect of SI on FS. In the first step, we fitted a model that included only a random intercept as the basis for our model comparison. We then fitted a model with the additivity factor. Note, the additivity factor is now defined slightly different than in section "Spatial and Temporal Contexts are Additive" (see main manuscript) and has five levels: SI baseline, SI baseline + 500 ms blank duration, SI baseline + 250 ms blank duration, SI baseline + 120 ms blank duration, and SI baseline + 0 ms blank duration – ordered from weakest to strongest effects. This model including the fixed effect of additivity factor, improved the fit compared to the random-intercept model significantly, LRT: $\chi^2(4) = 20.47$, $p < .001$, and showed a decrease in the information criterion $\Delta AIC = 12.47$.

Next, a model with a fixed effect of surround contrast was fitted. This also provided a significant increase in fit compared to the random intercept model, $\chi^2(1) = 82.17$, $p < .001$. A model including both surround contrast and the additivity factor, yielded a further increased fit (LRT: $\chi^2(4) = 21.07$, $p < .001$, $\Delta AIC = 80.77$) relative to the simple additivity factor model and also relative to the simple surround contrast model (LRT: $\chi^2(1) = 82.78$, $p < .001$, $\Delta AIC = 13.07$). Importantly, adding the interaction between the additivity factor and surround contrast further improved the model fit, $\chi^2(4) = 37.27$, $p < .001$. The interaction revealed that the influence of the prime was additive in the low-contrast condition but not in the high-contrast condition, as shown in Figure S1.

Figure S1

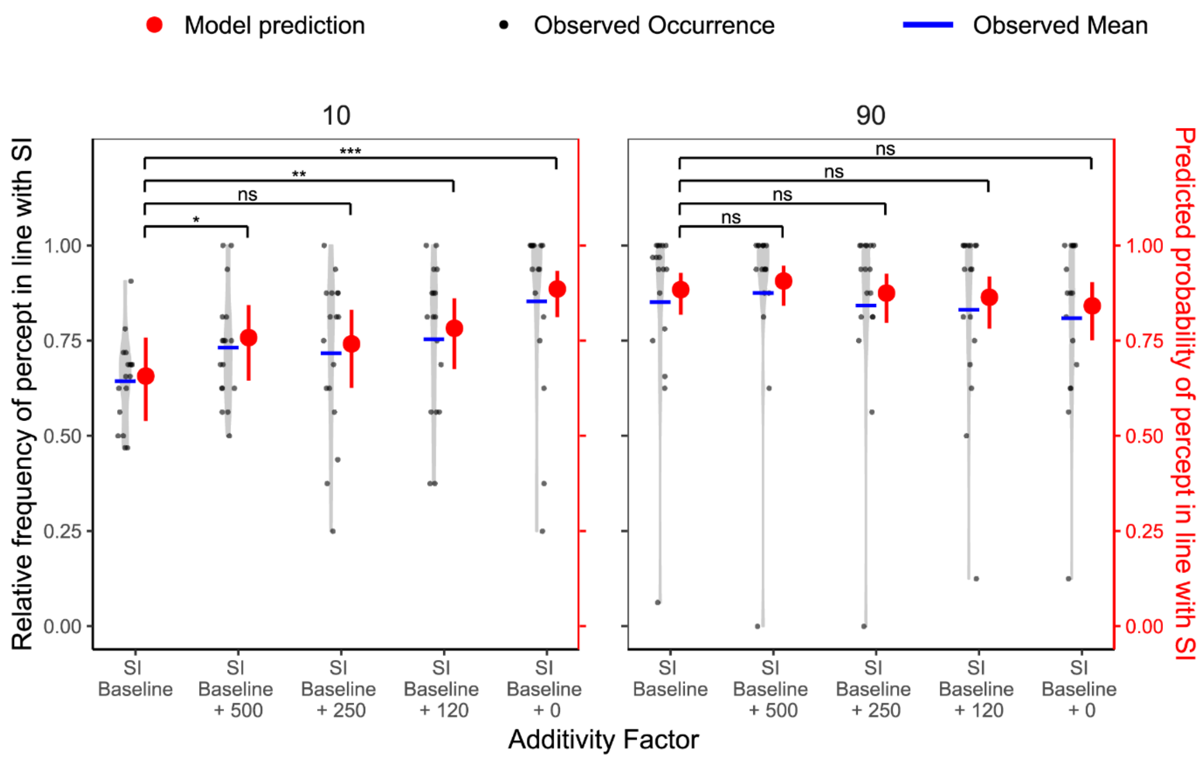

Additivity of blank duration and spatial context in Exp. 1. Relative frequency of the percept reported in line with the surround inhibition (SI) paradigm (black left y-axis) per participant and corresponding model predictions (red, right y-axis) across different levels of the additivity factor. The x-axis is ordered from weakest to strongest, starting with the pure SI baseline, followed by the SI-baseline with the FS conditions in increasing strength (i.e, decreasing blank duration (500 ms to 0 ms). Light gray violin plots represent the distribution of individual data, with jittered black dots indicating the observed values, and blue horizontal lines showing the observed condition means. Red points with error bars show model-predicted means and their 95% confidence intervals. Statistical significance is based on Bonferroni-corrected planned comparisons of model predictions; * = p < .05, ** = p < .01, *** = p < .001.

S.3 Clarity Ratings (Exp. 1)

The following Figure S2 shows the CLMM prediction for the clarity ratings in Exp. 1 for the competitive and additive conditions per surround contrast and blank duration. Similar to the 2AFC results, we see the strong contribution of surround contrast, i.e., drastically reduced ratings for low-contrast, compared to high-contrast surrounds. When the contrast is low, decreasing blank duration leads to higher clarity ratings.

Figure S2

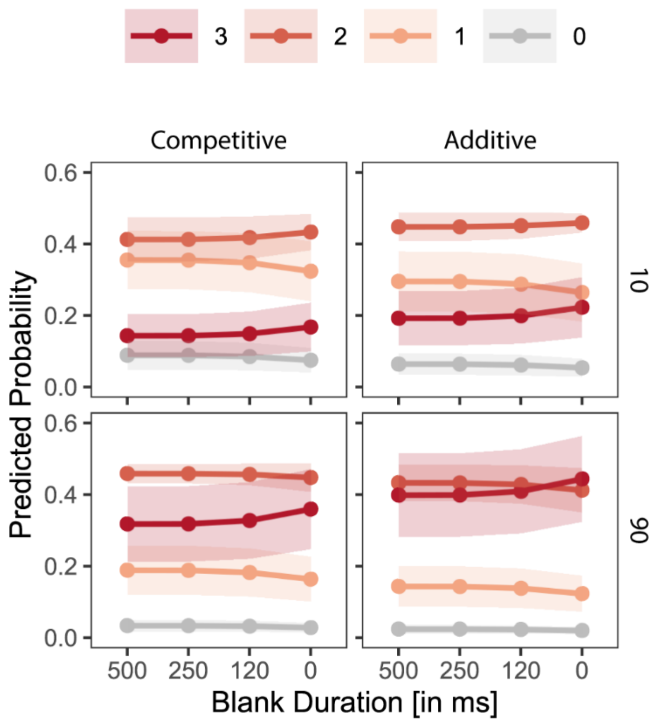

Results from clarity ratings in Exp. 1. Predicted probabilities of clarity ratings (0–3) from a Cumulative Link Mixed Model (CLMM), as a function of blank duration (x-axis), surround contrast (rows), and trial type (columns). Lines represent predicted probabilities for each rating level, with shaded ribbons indicating 95% confidence intervals. Higher values on the y-axis indicate greater model-predicted likelihood of a given clarity rating.

S.4 Interaction Probe Duration and Ocularity

To ensure that probe duration effects were not dependent on the ocularity of prime–probe presentation, we tested for an interaction between these two factors using a generalized linear mixed-effects model (GLMM) with a binomial link function. We compared a model that included main effects of probe duration and ocularity to a model that included their interaction. The likelihood ratio test showed that including the interaction did not significantly improve model fit, $\chi^2(3) = 6.38$, $p = .094$. While one interaction term (120 ms × same eye) reached significance individually ($p = .015$), the overall model comparison suggests no consistent or systematic interactions.

Supplement References

Bahmani, H., Li, Q., Logothetis, N. K., & Keliris, G. A. (2019). Responses of Neurons in Lateral Intraparietal Area Depend on Stimulus-Associated Reward During Binocular Flash Suppression. *Frontiers in Systems Neuroscience*, *13*(March), 1–12. https://doi.org/10.3389/fnsys.2019.00009

Bahmani, H., Murayama, Y., Logothetis, N. K., & Keliris, G. A. (2014). Binocular Flash Suppression in the Primary Visual Cortex of Anesthetized and Awake Macaques. *PLoS ONE*, *9*(9), e107628. https://doi.org/10.1371/journal.pone.0107628

Bartels, A., & Logothetis, N. K. (2010). Binocular rivalry: A time dependence of eye and stimulus contributions. *Journal of Vision*, *10*(12), 1–14. https://doi.org/10.1167/10.12.3

Brascamp, J. W., & Blake, R. (2012). Inattention Abolishes Binocular Rivalry. *Psychological Science*, *23*(10), 1159–1167. https://doi.org/10.1177/0956797612440100

Brascamp, J. W., Knapen, T. H. J., Kanai, R., van Ee, R., & van den Berg, A. V. (2007). Flash suppression and flash facilitation in binocular rivalry. *Journal of Vision*, *7*(12), 1–12. https://doi.org/10.1167/7.12.12

Gilroy, L. A., & Blake, R. (2005). The interaction between binocular rivalry and negative afterimages. *Current Biology*, *15*(19), 1740–1744. https://doi.org/10.1016/j.cub.2005.08.045

Holmes, D. J., Hancock, S., & Andrews, T. J. (2006). Independent binocular integration for form and colour. *Vision Research*, *46*(5), 665–677. https://doi.org/10.1016/j.visres.2005.05.023

Ikeda, K., & Morotomi, T. (2000). Feature-specific priming effects upon short duration binocular rivalry. *Psychologia*, *43*(2), 123–134.

Ikeda, K., & Morotomi, T. (2002). Color-specific filtering of rival binocular inputs induced by priming. *Japanese Psychological Research*, *44*(2), 57–65. https://doi.org/10.1111/1468-5884.00007

Ikeda, K., & Morotomi, T. (2007). Alteration ofinterocular suppression following monocular homogeneous stimulus. *Psychologia*, *50*(1), 47–53. https://doi.org/10.2117/psysoc.2007.47

Ikeda, K., Ogata, Y., & Morotomi, T. (2009). Perceptual experience and temporal two stages in flash suppression for contour rivalry. *Psychologia*, *52*(1), 25–40. https://doi.org/10.2117/psysoc.2009.25

Kanai, R., Walsh, V., & Tseng, C. H. (2010). Subjective discriminability of invisibility: A framework for distinguishing perceptual and attentional failures of awareness. *Consciousness and Cognition*, *19*(4), 1045–1057. https://doi.org/10.1016/j.concog.2010.06.003

Kapoor, V., Besserve, M., Logothetis, N. K., & Panagiotaropoulos, T. I. (2018). Parallel and functionally segregated processing of task phase and conscious content in the prefrontal cortex. *Communications Biology*, *1*(1). https://doi.org/10.1038/s42003-018-0225-1

Kapoor, V., Dwarakanath, A., Safavi, S., Werner, J., Besserve, M., Panagiotaropoulos, T. I., & Logothetis, N. K. (2022). Decoding internally generated transitions of conscious contents in the prefrontal cortex without subjective reports. *Nature Communications*, *13*(1), 1535. https://doi.org/10.1038/s41467-022-28897-2

Keliris, G. A., Logothetis, N. K., & Tolias, A. S. (2010). The Role of the Primary Visual Cortex in Perceptual Suppression of Salient Visual Stimuli. *The Journal of Neuroscience*, *30*(37), 12353–12365. https://doi.org/10.1523/JNEUROSCI.0677-10.2010

Kreiman, G., Fried, I., & Koch, C. (2002). Single-neuron correlates of subjective vision in the human medial temporal lobe. *Proceedings of the National Academy of Sciences*, *99*(12), 8378–8383. https://doi.org/10.1073/pnas.072194099

Ling, S., & Blake, R. (2010). Suppression during binocular rivalry broadens orientation tuning. *Journal of Vision*, *8*(6), 246–246. https://doi.org/10.1167/8.6.246

Maier, A., Logothetis, N. K., & Leopold, D. A. (2007). Context-dependent perceptual modulation of single neurons in primate visual cortex. *Proceedings of the National Academy of Sciences*, *104*(13), 5620–5625. https://doi.org/10.1073/pnas.0608489104

Meng, M., Ferneyhough, E., & Tong, F. (2007). Dynamics of perceptual filling-in of visual phantoms revealed by binocular rivalry. *Journal of Vision*, *7*(13), 1–15. https://doi.org/10.1167/7.13.8

Moradi, F., & Heeger, D. J. (2009). Inter-ocular contrast normalization in human visual cortex. *Journal of Vision*, *9*(3), 13–13. https://doi.org/10.1167/9.3.13

Nichols, D. F., & Wilson, H. R. (2009). Effect of transient versus sustained activation on interocular suppression. *Vision Research*, *49*(1), 102–114. https://doi.org/10.1016/j.visres.2008.09.033

Ooi, T. L., & Loop, M. S. (1994). Visual suppression and its effect upon color and luminance sensitivity. *Vision Research*, *34*(22), 2997–3003. https://doi.org/10.1016/0042-6989(94)90272-0

Palmer, T. D., & Ramsey, A. K. (2012). The function of consciousness in multisensory integration. *Cognition*, *125*(3), 353–364. https://doi.org/10.1016/j.cognition.2012.08.003

Panagiotaropoulos, T. I., Deco, G., Kapoor, V., & Logothetis, N. K. (2012). Neuronal Discharges and Gamma Oscillations Explicitly Reflect Visual Consciousness in the Lateral Prefrontal Cortex. *Neuron*, *74*(5), 924–935. https://doi.org/10.1016/j.neuron.2012.04.013

Parker, A., & Alais, D. (2007). A bias for looming stimuli to predominate in binocular rivalry. *Vision Research*, *47*(20), 2661–2674. https://doi.org/10.1016/j.visres.2007.06.019

Pearson, J., & Clifford, C. W. G. (2005a). Mechanisms selectively engaged in rivalry: normal vision habituates, rivalrous vision primes. *Vision Research*, *45*(6), 707–714. https://doi.org/10.1016/j.visres.2004.09.040

Pearson, J., & Clifford, C. W. G. (2005b). Suppressed Patterns Alter Vision during Binocular Rivalry.

Current Biology, *15*(23), 2142–2148. https://doi.org/10.1016/j.cub.2005.10.066

Sheinberg, D. L., & Logothetis, N. K. (1997). The role of temporal cortical areas in perceptual organization. *Proceedings of the National Academy of Sciences*, *94*(7), 3408–3413. https://doi.org/10.1073/pnas.94.7.3408

Tsuchiya, N., Koch, C., Gilroy, L. A., & Blake, R. (2006). Depth of interocular suppression associated with continuous flash suppression, flash suppression, and binocular rivalry. *Journal of Vision*, *6*(10), 1068–1078. https://doi.org/10.1167/6.10.6

van Ee, R. (2011). Percept-switch nucleation in binocular rivalry reveals local adaptation characteristics of early visual processing. *Journal of Vision*, *11*(2), 1–12. https://doi.org/10.1167/11.2.1

Wolfe, J. M. (1984). Reversing ocular dominance and suppression in a single flash. *Vision Research*, *24*(5), 471–478. https://doi.org/10.1016/0042-6989(84)90044-0
